# Beyond Weight Reduction: An Updated Systematic Review and Meta-Analysis of Metabolic Syndrome Components and Metabolic Dysfunction-Associated Steatotic Liver Disease Outcomes After Endoscopic Sleeve Gastroplasty

**DOI:** 10.3390/jcm15176633

**Published:** 2026-08-27

**Authors:** Alexander Herner, Simon Nennstiel, Christoph Schlag, Andreas Kremer, Luc Biedermann, Gerhard Rogler, Michael Doulberis

**Affiliations:** Department of Gastroenterology and Hepatology, University Hospital Zurich, University of Zurich, 8091 Zurich, Switzerland

**Keywords:** endoscopic sleeve gastroplasty, metabolic syndrome, metabolic dysfunction-associated steatotic liver disease, bariatric endoscopy, weight loss

## Abstract

**Background**: Endoscopic sleeve gastroplasty (ESG) is a minimally invasive option for weight reduction, but its effect on metabolic syndrome (MetS) and metabolic dysfunction-associated steatotic liver disease (MASLD) is less well characterized. **Methods**: Following PRISMA, we searched five databases to November 2025 (PROSPERO CRD420261283041) for adults with obesity undergoing ESG with ≥6-month follow-up. Continuous outcomes were pooled as mean differences (MDs) using DerSimonian–Laird random-effects models with Hartung–Knapp–Sidik–Jonkman sensitivity analysis. As MetS resolution was inconsistently defined, the primary outcome was post hoc operationalised as change in individual MetS components. **Results**: Of 858 records, 12 studies (4452 patients; two randomised) were included. At 12 months, ESG was associated with lower glycated haemoglobin (MD −0.33%), HOMA-IR (MD −3.52), triglycerides (MD −25.56 mg/dL), low-density lipoprotein cholesterol (MD −6.94 mg/dL), alanine aminotransferase, and non-invasive steatosis/fibrosis indices; aspartate aminotransferase did not change significantly. Total body weight loss was 16.54% at 12 months and 14.21% at 36 months. Liver stiffness (two studies, differing windows) is described narratively, not pooled. Heterogeneity was often substantial; certainty was low to very low. **Conclusions**: ESG was associated with metabolic and hepatic improvements alongside substantial weight loss; a weight-independent hepatic effect cannot be established, and randomised trials with histological endpoints are needed.

## 1. Introduction

Visceral obesity and metabolic dysfunction are acknowledged as a major global public-health burden. In 2022, an estimated 43% of adults globally were considered to be overweight, and 16% were obese, where adult obesity has more than doubled since 1990 [1]. Within Europe, almost 60% of adults be overweight or have obesity [2], and in Switzerland approximately 43% of citizens aged 15 years or older are overweight or have obesity, of whom about 12% have obesity [3].

In parallel, the metabolic syndrome (MetS) remains highly prevalent. According to the International Diabetes Federation definition, MetS is present when central obesity (waist circumference ≥ 94 cm for men and ≥80 cm for women in Europeans) is accompanied by any two of raised fasting glucose or diabetes, elevated triglycerides, lowered high-density lipoprotein (HDL) cholesterol, or elevated arterial blood pressure. This constellation reflects the clustering of insulin resistance, arterial hypertension, dyslipidaemia, and visceral adiposity, which raises cardiometabolic and liver risk [4]. Of note, about 25–30% of European adults meet the aforementioned criteria [5,6]. Given its mechanistic links with hepatic insulin resistance and steatosis, MetS is recognised as a systemic driver of metabolic dysfunction-associated steatotic liver disease (MASLD, previously non-alcoholic fatty liver disease), the hepatic manifestation of MetS [5,7].

Metabolic and bariatric surgery, principally laparoscopic sleeve gastrectomy (LSG) and Roux-en-Y gastric bypass, achieves substantial and often durable weight loss, but its usage is restricted by surgical risk, anatomical irreversibility, peri-operative complications, and resource demands [8]. Long-term data suggest that survival in this population remains reduced relative to the general population despite surgery [9]. Moreover, altered anatomy after Roux-en-Y gastric bypass may complicate subsequent liver transplantation and the endoscopic management of biliary complications, an additional consideration in patients with advanced MASLD-related fibrosis [10,11].

Endoscopic sleeve gastroplasty (ESG) has emerged as a minimally invasive, organ-sparing endoscopic variant; firstly described in its current form about 2013, it has accumulating evidence for safety and effectiveness [12]. Systematic reviews, comparative analyses, and position statements report that ESG provides meaningful weight loss with considerably lower serious adverse-event rates than surgical alternatives [13,14,15].

Despite multiple meta-analyses of ESG, methodological gaps persist: to these belong heterogeneous inclusion criteria, variable follow-up, sparse reporting of liver-specific surrogates, and limited adjustment for concomitant pharmacotherapy. In particular, the most closely related prior synthesis, Hanscom et al. [16], pooled 10 studies (4320 patients) through an earlier search date. Relative to that review, the present analysis incorporates three studies not previously synthesised: Abad et al. [17], the only sham-controlled randomised trial with paired liver-stiffness data; Jirapinyo et al. [18], from which only the endoscopic sleeve gastroplasty monotherapy arm was extracted; and Hajifathalian et al. [19], an early report from the same registry as Lahooti et al. [20] and treated accordingly as a partially overlapping cohort (Section 2). Beyond these two additional independent cohorts, the present review extends Hanscom et al. [16] methodologically, applying Hartung–Knapp–Sidik–Jonkman inference, an imputed within-subject correlation sensitivity analysis, ROBINS-I/RoB 2 risk-of-bias assessment, and GRADE certainty rating, and adds longer-term (36-month) weight data and a dedicated synthesis of MASLD-related hepatic outcomes; as detailed in the Section 5, the additional studies did not materially change the metabolic conclusions relative to Hanscom et al. [16]. We therefore conducted an updated systematic review and meta-analysis. The pre-specified primary objective was to assess change in MetS following ESG; because complete MetS resolution could not be pooled reliably (Section 2), this objective was operationalised post hoc as change in individual MetS components, with secondary objectives of assessing weight-related and liver-related parameters.

## 2. Materials and Methods

### 2.1. Protocol and Registration

This systematic review and meta-analysis was performed in accordance with the Preferred Reporting Items for Systematic Reviews and Meta-Analyses (PRISMA) 2020 statement [21] and the Cochrane Handbook for Systematic Reviews of Interventions (version 6.5, August 2024) [22]. The protocol was registered in the International Prospective Register of Systematic Reviews [23] (PROSPERO; CRD420261283041). As only previously published, de-identified data were used, institutional review board approval was waived.

### 2.2. Research Question and Eligibility

The review followed the Population, Intervention, Comparison, Outcome (PICO) framework [24]. Population: adults (≥18 years) with obesity who underwent ESG, among whom metabolic and/or hepatic outcomes were evaluated; individual studies applied differing eligibility thresholds, most commonly BMI ≥ 30 kg/m^2^ alone or a lower threshold (BMI ≥ 26–27.5 kg/m^2^) permitted when obesity-related comorbidities such as hypertension, type 2 diabetes, or dyslipidaemia were present, and one study additionally enrolled surgery-eligible patients who declined surgery regardless of BMI; studies also differed in whether MASLD or specific metabolic comorbidities were required for enrolment versus assessed only as outcomes, contributing to clinical heterogeneity in the pooled population (full eligibility criteria by study are given in Supplementary Table S1). Intervention: ESG with the Apollo OverStitch system or an equivalent endoscopic suturing system. Comparison: baseline versus post-ESG follow-up values; where controlled studies were available, between-group comparisons were considered separately. The primary quantitative synthesis pools within-group (pre-post) changes in the ESG arm; only two of the twelve included studies had a randomised comparator, and pooled estimates are therefore reported as changes observed after ESG rather than effects causally attributable to ESG. Between-group findings from the two randomised trials are summarised narratively in the Section 4 (Supplementary Table S2).

Outcomes: The pre-specified primary outcome was change in MetS following ESG. Because complete MetS resolution was inconsistently defined and reported, with individual studies drawing on differing diagnostic frameworks (e.g., ATP III, AHA/NHLBI, or IDF criteria) or not specifying a formal definition at all, and could not be operationalised for pooling, the primary analysis was operationalised post hoc as changes in individual MetS components, including glycaemic status, lipid fractions, and blood pressure. Only studies with at least six months of follow-up were eligible.

### 2.3. Search Strategy and Study Selection

A systematic search was performed across PubMed (MEDLINE), Embase, Scopus, Cochrane, and Web of Science from inception to November 2025, with no date limits; only peer-reviewed English-language articles were eligible. The strategy combined controlled vocabulary and free-text terms for ESG and metabolic or hepatic outcomes (Supplementary Table S3). Records were imported into EndNote 21 for Mac. Two reviewers (AH, MD) independently screened titles, abstracts, and full texts; discrepancies were resolved by consensus or by a third senior reviewer (GR). When a group reported multiple publications, only the most recent and complete report was retained. Because Hajifathalian et al. [19] and Lahooti et al. [20] originated from the same institution and senior author, with overlapping recruitment windows (August 2013–August 2019 and August 2013–June 2024, respectively), they were treated as reporting a shared, nested patient cohort; Lahooti et al. [20], as the larger and more recent report spanning the earlier enrolment period, was retained as the primary source, and Hajifathalian et al. [19] was excluded from the two pooled outcomes it shares with Lahooti et al. [20] (homeostatic model assessment of insulin resistance and alanine aminotransferase) to avoid double-counting of overlapping participants. Corresponding authors were contacted for missing data. Data extraction also captured, where reported, the post-procedural dietary and exercise programme, dietitian or multidisciplinary follow-up, adherence assessment, and changes in glucose-lowering, lipid-lowering, and weight-loss medications, summarised in Supplementary Table S1.

### 2.4. Risk of Bias and Certainty of Evidence

Risk of bias was assessed with the Risk Of Bias In Non-randomised Studies of Interventions (ROBINS-I) tool for non-randomised studies and the Risk of Bias 2 (RoB 2) tool for randomised trials [25,26]. Certainty of evidence was rated as high, moderate, low, or very low using the Grading of Recommendations Assessment, Development and Evaluation (GRADE) approach (GRADEpro Guideline Development Tool) [27].

### 2.5. Statistical Analysis

Statistical analyses focused on continuous outcomes. For each parameter, the mean difference (MD) between baseline and follow-up was calculated with 95% confidence intervals (CIs). Throughout the manuscript, tables, and figures, MD is defined and reported consistently as follow-up minus baseline, such that a negative value indicates a decrease and a positive value indicates an increase after ESG. For studies reporting separate baseline and follow-up values, the MD was calculated directly; for studies reporting only mean change, the generic inverse-variance method was used, with the standard error derived from the reported standard deviation or imputed using a correlation coefficient of 0.5 when unavailable, as recommended by the Cochrane Handbook [22]. Values reported as a median with interquartile range were treated as a mean with standard deviation only where the reported timepoints indicated a symmetrical distribution; this applied to the triglyceride values of Espinet-Coll et al. [28] and is a conservative treatment because an interquartile range exceeds the corresponding standard deviation and therefore reduces rather than increases that study’s weight. Where a median-reported value could not be distinguished from the value printed for an adjacent row of the same table, it was not used at all. Where separate baseline and follow-up values were used, the baseline mean and sample size reflect the originally treated or enrolled cohort as reported by the primary study, not a value recalculated among those who later completed follow-up, because individual patient data were not available; the follow-up sample size reflects participants with a measurement at that time point, and the standard error was estimated using the standard formula for two independent samples, which is a conservative approach that does not incorporate the within-subject correlation between the two measurements. Per-outcome baseline and follow-up sample sizes and attrition are reported in Section 4. A sensitivity analysis re-estimating these outcomes using an imputed within-subject correlation is described below. Because only two included studies were randomised, a formal between-group meta-analysis was not feasible; the primary synthesis therefore describes within-group change in the ESG arm, and between-group results reported by the randomised trials themselves are presented narratively rather than pooled. Time windows for pooling were prespecified as follows: 6-month bucket, 4–8 months; 12-month bucket, 9–15 months; 24-month bucket, 20–28 months; 36-month bucket, 30–42 months. Where a study’s reported assessment for a given outcome fell outside the applicable window, that study was excluded from the corresponding pooled analysis, and its result was reported narratively instead; leave-one-out and time-window exclusions are distinguished from the single-cohort-exclusion and paired-correlation sensitivity analyses described below.

The primary analysis used a random-effects model with DerSimonian–Laird estimation of the between-study variance [29]. Because several outcomes included few studies, where the normal-based interval can be anticonservative, we additionally applied a Hartung–Knapp–Sidik–Jonkman (HKSJ) adjustment as a sensitivity analysis; this derives confidence intervals from a t-distribution with k − 1 degrees of freedom and yields better-calibrated intervals when studies are few. Effect sizes are reported with confidence intervals rather than relying on *p* values alone; a two-sided *p* value < 0.05 was considered significant, and heterogeneity was quantified with the I^2^ statistic. Because few outcomes included 10 or more studies, formal tests for funnel-plot asymmetry were generally not performed. A funnel plot was constructed for the best-populated outcome (Supplementary Figure S1), but publication bias and small-study effects could not be assessed reliably because of the limited number of studies and substantial heterogeneity. Two further post hoc sensitivity analyses were performed: leave-one-out removal of individual studies, and exclusion of the cohort reported by Jirapinyo et al. [18], the only included cohort drawn from a study of ESG combined with a glucagon-like peptide-1 (GLP-1) receptor agonist. From that study only the endoscopic monotherapy arm was extracted, so no pooled estimate in this review includes patients receiving a GLP-1 receptor agonist; this analysis therefore examines whether the smallest and most highly selected cohort, comprising patients with compensated advanced chronic liver disease, influenced the pooled estimates. Analyses were performed in Review Manager (RevMan) version 5.4 (The Cochrane Collaboration, London, UK); the HKSJ sensitivity analysis was additionally computed with the metafor package for R (version 4.3.2).

## 3. Ethics

This study used only previously published, de-identified data and was therefore exempt from any institutional ethics approval. No financial compensation or industry support influenced study design or data interpretation.

## 4. Results

### 4.1. Study Selection and Characteristics

**Figure 1:**
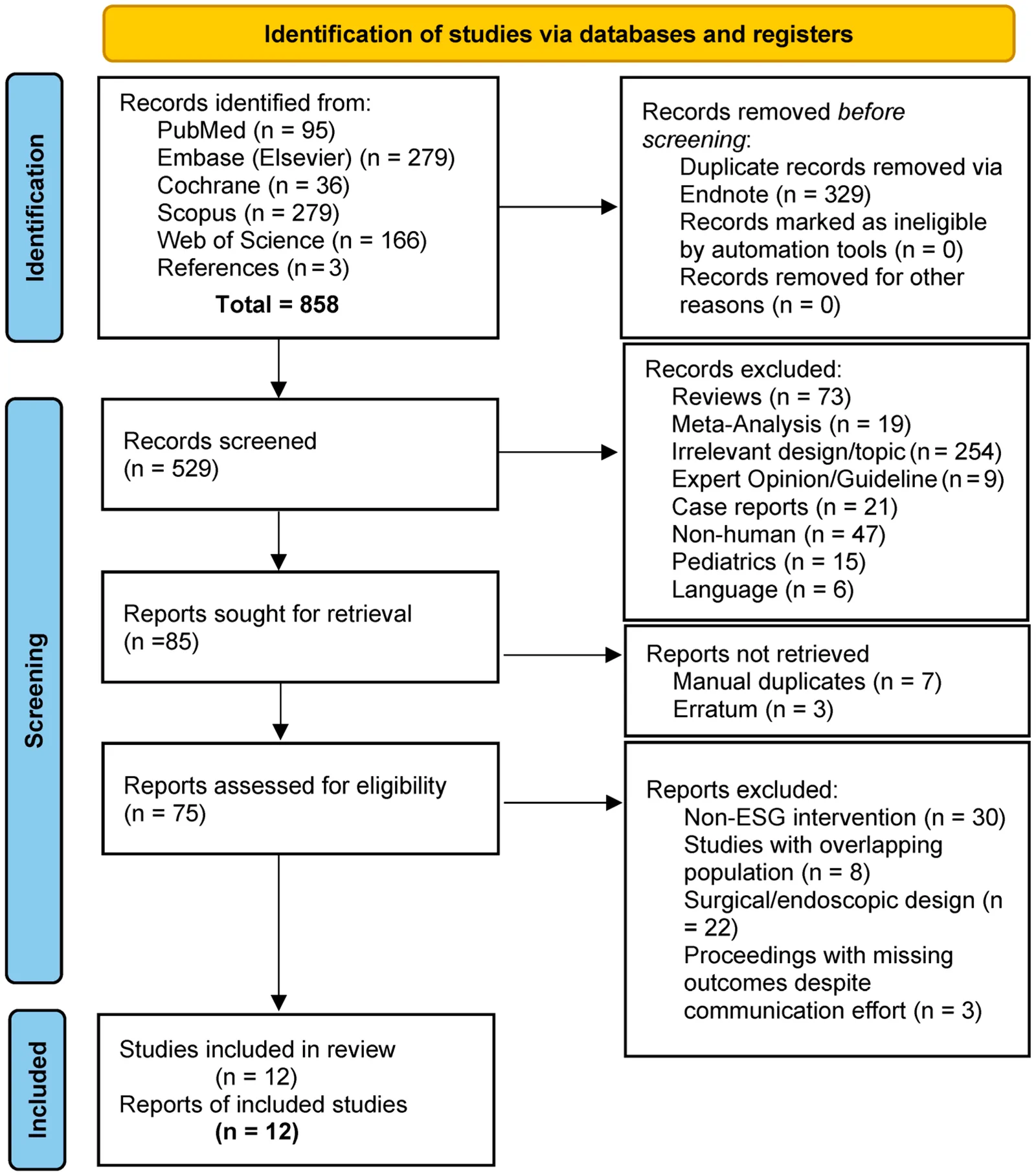
PRISMA 2020 flow diagram of study selection for the systematic review and meta-analysis on endoscopic sleeve gastroplasty (ESG) and metabolic/steatohepatitis-related outcomes. A total of 858 records were identified across databases and reference screening; after removal of 329 duplicates, 529 records were screened. Seventy-five full texts were assessed for eligibility, and 12 studies were included in the final qualitative and quantitative synthesis.

Of 858 records identified, 529 remained after deduplication; 444 were excluded at title and abstract screening, 85 reports were sought for retrieval (10 of which were not retrieved: seven manual duplicates and three errata) and 75 underwent full-text assessment, leaving 12 reports that met the inclusion criteria [17,18,19,20,28,30,31,32,33,34,35,36] (Figure 1). Baseline characteristics are in Table 1 and baseline laboratory values in Supplementary Table S4. Eligibility criteria, liver-disease definitions, and reported co-interventions for each study are summarised in Supplementary Table S1. Two reports were randomised [17,30]. A total of 4452 patients were included; the pooled mean age was 36.9 years, the weighted baseline BMI was 33.8 kg/m^2^, and 83.5% of participants were women. Most studies were small, with two exceptions [32,34]. Consistent with the six-month minimum follow-up eligibility criterion (Section 2), follow-up available for pooling ranged from 6 months to 5 years, with 12 months the most common interval; some studies additionally reported earlier interim assessments (e.g., 3 months) that were not used in the pooled analyses. Data availability per outcome is summarised in Table 2.

### 4.2. Metabolic Syndrome Components

**Figure 2:**
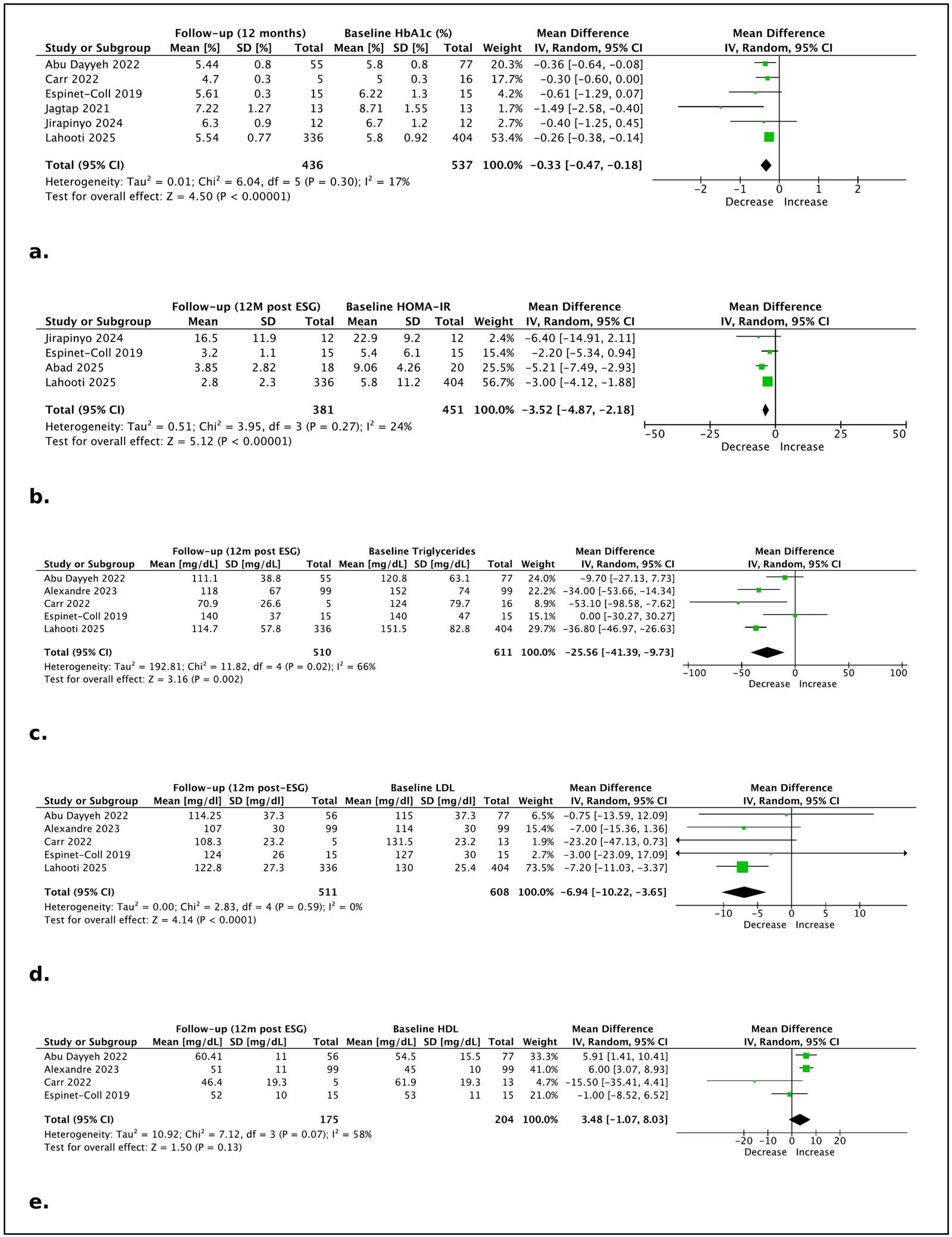
Impact of ESG on metabolic syndrome components at 12 months. Forest plots demonstrating the mean difference (MD, follow-up minus baseline; negative values indicate a decrease) at 12-month follow-up for: (**a**) HbA1c (%): Significant pooled MD −0.33% [95% CI: −0.47, −0.18]. (**b**) HOMA-IR: Significant pooled MD −3.52 [95% CI: −4.87, −2.18] (four studies; Hajifathalian et al. [19] excluded because of cohort overlap with Lahooti et al. [20], see Section 2). (**c**) Triglycerides (mg/dL): Significant pooled MD −25.56 mg/dL [95% CI: −41.39, −9.73], with substantial heterogeneity (I^2^ = 66%). (**d**) LDL Cholesterol (mg/dL): Significant pooled MD −6.94 mg/dL [95% CI: −10.22, −3.65]. (**e**) HDL Cholesterol (mg/dL): Non-significant pooled MD +3.48 mg/dL [95% CI: −1.07, 8.03], i.e., a small increase that does not reach significance. Note: Markers represent the mean difference (follow-up minus baseline); negative values indicate a decrease and positive values an increase after ESG. Horizontal lines represent 95% confidence intervals (CIs). The diamond at the bottom of each plot represents the pooled effect size. ESG, endoscopic sleeve gastroplasty; IV, inverse variance. Studies contributing to each panel: (**a**) [18,20,28,30,35,36]; (**b**) [17,18,20,28]; (**c**) [20,28,30,31,35]; (**d**) [20,28,30,31,35]; (**e**) [28,30,31,35].

For glycaemic control, the pooled analysis (537 patients) showed a change in glycated haemoglobin of MD −0.33% (95% CI −0.47 to −0.18; *p* < 0.001) at 12 months, with low heterogeneity (I^2^ = 17%). The homeostatic model assessment of insulin resistance, excluding Hajifathalian et al. [19] because of its cohort overlap with Lahooti et al. [20] (Section 2), changed by MD −3.52 (95% CI −4.87 to −2.18; *p* < 0.001; four studies; I^2^ = 24%). For lipids, ESG was associated with a reduction in triglycerides (MD −25.56 mg/dL; 95% CI −41.39 to −9.73; *p* = 0.002; I^2^ = 66%) and low-density lipoprotein cholesterol (MD −6.94 mg/dL; 95% CI −10.22 to −3.65; *p* < 0.001; I^2^ = 0%). HDL cholesterol showed a non-significant increase (MD 3.48 mg/dL; 95% CI −1.07 to 8.03; *p* = 0.13; I^2^ = 58%) (Figure 2). For arterial hypertension, most studies reported a reduction in prevalence or antihypertensive burden at follow-up, but standardised longitudinal data were too limited for reliable pooling.

### 4.3. Hepatic Parameters

**Figure 3:**
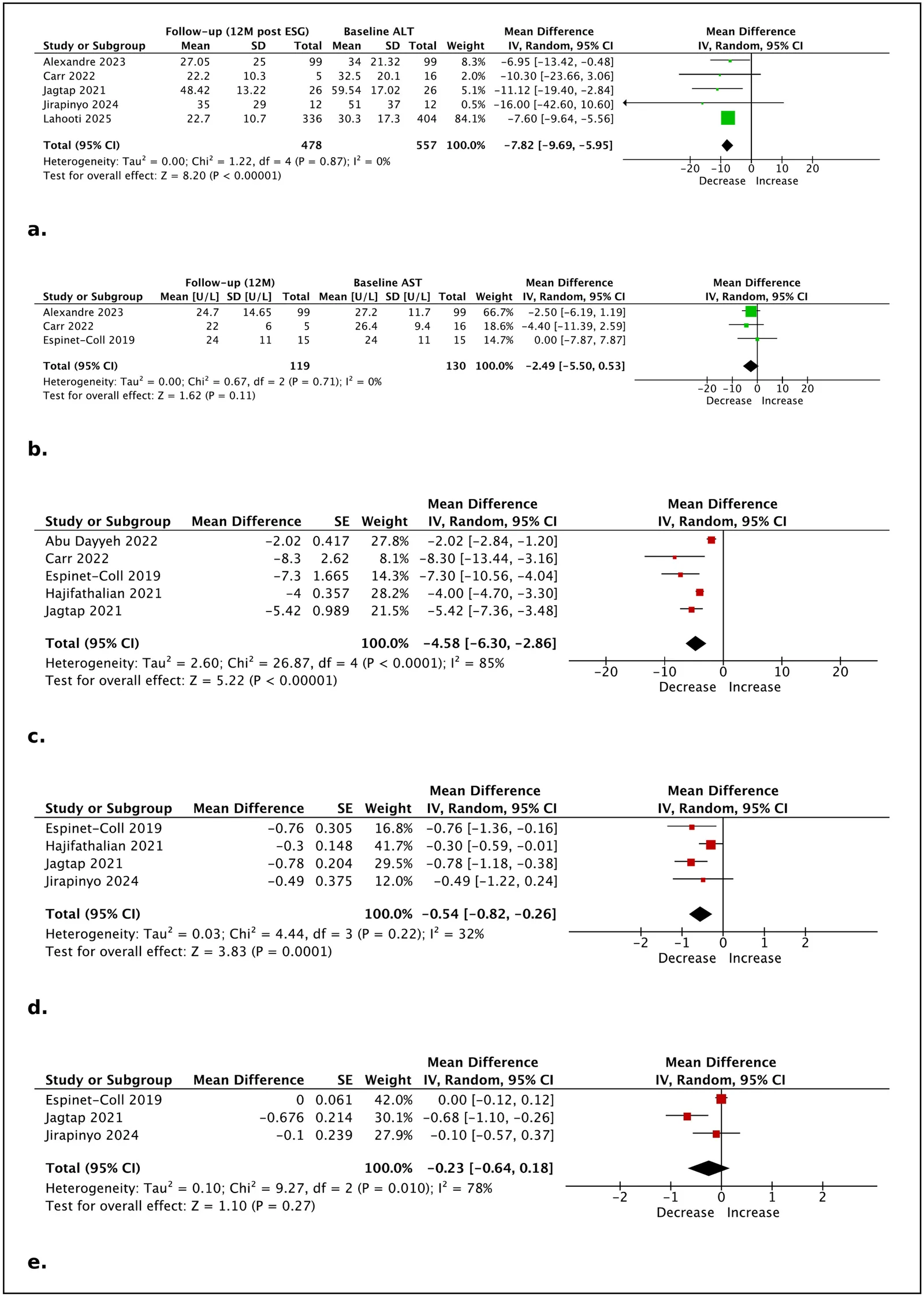
Forest plots of hepatic outcomes 12 months after endoscopic sleeve gastroplasty (ESG). The plots illustrate the changes between baseline and 12-month follow-up for liver-related biochemical and non-invasive markers. ALT excludes Abad et al. [17], Hajifathalian et al. [19] and Espinet-Coll et al. [28]; AST excludes Abad et al. [17], Lahooti et al. [20], Jagtap et al. [36] and Jirapinyo et al. [18] (time-window, cohort-overlap, and data-availability criteria, see Section 2). (**a**) Alanine aminotransferase (ALT): Significant reduction in serum ALT levels, a biochemical marker of hepatocellular injury. (**b**) Aspartate aminotransferase (AST): Change in AST levels following the procedure; the reduction did not reach statistical significance. (**c**) Hepatic Steatosis Index (HSI): Significant reduction in the HSI score; HSI incorporates BMI and should not be interpreted as direct evidence of reduced hepatic fat. (**d**) NAFLD Fibrosis Score (NFS): Significant reduction in the NFS score; NFS also incorporates BMI and metabolic variables and should not be interpreted as direct evidence of fibrosis improvement. (**e**) FIB-4 index: Change in the FIB-4 index; the reduction did not reach statistical significance at the 12-month mark. Liver stiffness measurement (LSM) by transient elastography is not displayed as a panel; it was reported by only two studies (30 patients) at genuinely different follow-up windows and was therefore not pooled, and is described narratively in the Section 4; given this very small and heterogeneous sample, the finding should be regarded as exploratory and does not establish regression of hepatic fibrosis. The diamond at the bottom of each plot represents the pooled effect size. A random-effects model was applied for the pooled analyses. These panels depict changes in surrogate biochemical and non-invasive indices, not histological outcomes; HSI and NFS are composite indices that incorporate BMI and other metabolic variables and may decline partly as a mathematical consequence of weight loss rather than a direct hepatic effect. Findings should not be interpreted as evidence of reduced hepatic inflammation, reduced liver fat, or fibrosis regression. ALT, alanine aminotransferase; AST, aspartate aminotransferase; CI, confidence interval; ESG, endoscopic sleeve gastroplasty; FIB-4, fibrosis-4 index; HSI, Hepatic Steatosis Index; kPa, kilopascals; LSM, liver stiffness measurement; MD, mean difference; NFS, NAFLD Fibrosis Score. Studies contributing to each panel: (**a**) [18,20,31,35,36]; (**b**) [28,31,35]; (**c**) [19,28,30,35,36]; (**d**) [18,19,28,36]; (**e**) [18,28,36].

ESG was associated with lower alanine aminotransferase (ALT) at 12 months, whereas the change in aspartate aminotransferase (AST) did not reach statistical significance. Because the sham-controlled trial of Abad et al. [17] assessed ALT and AST at week 72 (~16.6 months) rather than within the prespecified 9–15 month window for the 12-month analysis, it was excluded from both pooled estimates (see Section 2) and is instead reported narratively below; the same criterion excluded this trial from the 12-month total body weight-loss analysis (Section 4, Weight loss). Among the remaining five studies (additionally excluding Hajifathalian et al. [19] because of its cohort overlap with Lahooti et al. [20], and Espinet-Coll et al. [28], whose ESG-arm ALT values were reported as a median with interquartile range and could not be distinguished from those printed for the adjacent gamma-glutamyl transferase row; Section 2), ALT changed by MD −7.82 U/L (95% CI −9.69 to −5.95; *p* < 0.001; 557 patients), with no heterogeneity (I^2^ = 0%). For AST, Lahooti et al. [20], Jagtap et al. [36] and Jirapinyo et al. [18] did not report post-procedural AST values and could not contribute to this pooled estimate (Supplementary Table S4); post-ESG AST at 12 months was therefore available from only three studies, in which AST changed by MD −2.49 U/L (95% CI −5.50 to 0.53; *p* = 0.11; 130 patients), with no detectable heterogeneity for either outcome (I^2^ = 0%). In the sham-controlled trial, mean ALT and AST changes from baseline to week 72 were −27.67 U/L and −21.33 U/L, respectively, in the ESG group versus −13.0 U/L and −12.22 U/L in the sham group. Non-invasive scores also changed: the Hepatic Steatosis Index MD was −4.58 points (95% CI −6.30 to −2.86; *p* < 0.001), and the NAFLD Fibrosis Score MD was −0.54 (95% CI −0.82 to −0.26; *p* < 0.001); the Fibrosis-4 index showed a non-significant change (MD −0.23; 95% CI −0.64 to 0.18). Liver stiffness measurement by transient elastography (Fibroscan) was reported by only two studies at genuinely different follow-up windows (Jirapinyo et al. [18], 12 months, n = 12; Abad et al. [17], week 72 [~16.6 months], n = 18) and is therefore reported narratively rather than pooled, consistent with the time-window criterion applied to ALT and AST above. Jirapinyo et al. [18] reported a mean change of −1.8 kPa (SE 3.78) at 12 months; Abad et al. [17] reported a mean change of −5.63 kPa (SD 7.2) in the ESG group versus −0.2 kPa (SD 5.38) in the sham group at week 72 (*p* = 0.017). These small, heterogeneous, non-poolable data should not be interpreted as evidence of fibrosis regression (Figure 3).

### 4.4. Weight Loss

**Figure 4:**
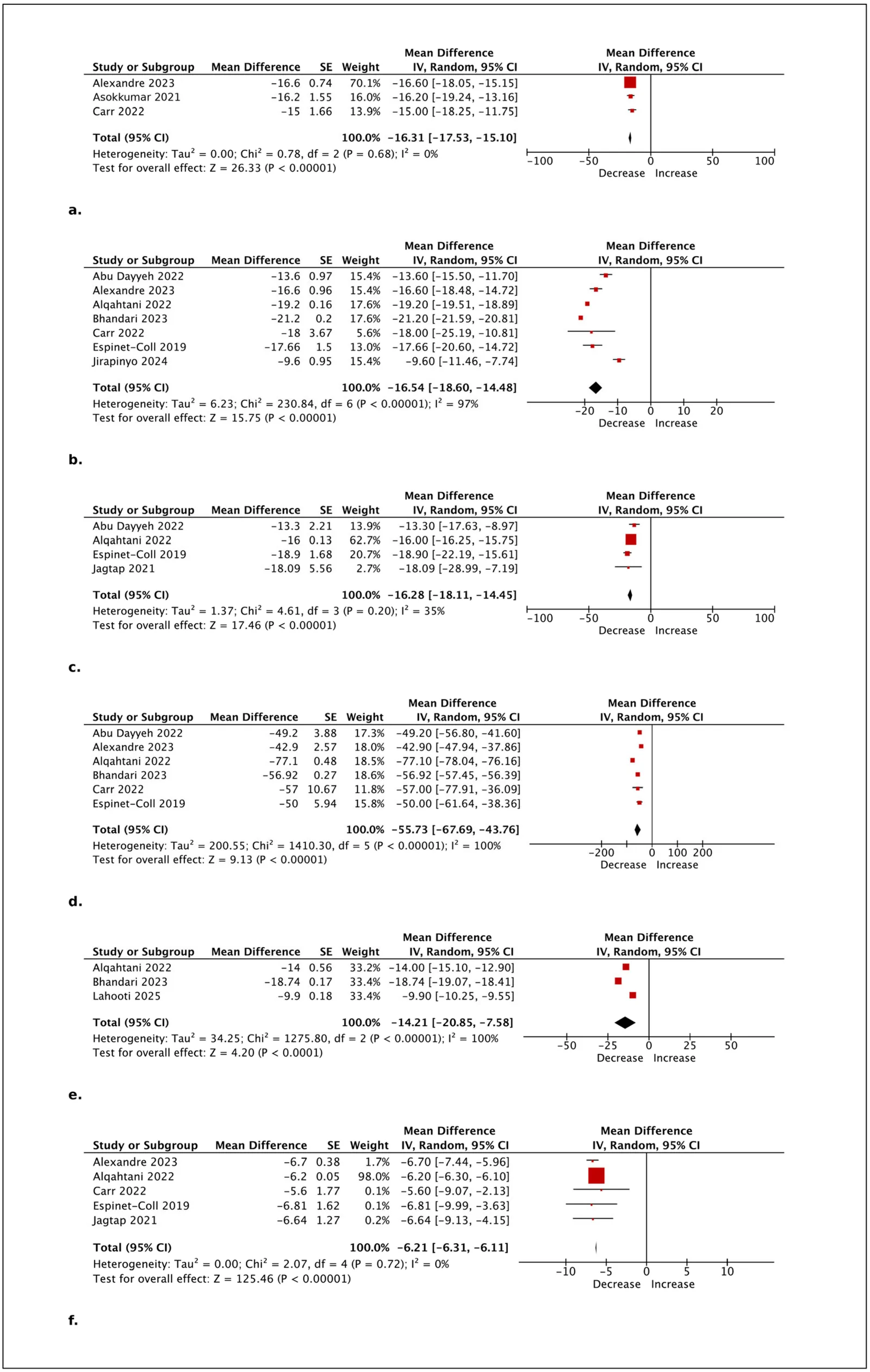
The plots show pooled weight loss at each available follow-up interval, using various standardised metrics. Because the 6-, 12-, and 36-month estimates are drawn from partly non-overlapping sets of studies (Table 2), they represent heterogeneous cross-sectional summaries rather than a single cohort’s trajectory over time (see Section 5). (**a**) %Total Body Weight Loss (%TBWL) at 6 months: Early significant weight reduction observed within the first half-year post-procedure. (**b**) %Total Body Weight Loss (%TBWL) at 12 months: Pooled weight loss observed across the contributing studies at 12 months. (**c**) Absolute Weight Loss (kg) at 12 months: Significant decrease in total body weight measured in kilograms. (**d**) %Excess Weight Loss (%EWL) at 12 months: Significant reduction in excess body weight, confirming the clinical impact on obesity. (**e**) %Total Body Weight Loss (%TBWL) at 36 months: Weight loss remained observable in the limited number of cohorts (three studies) contributing longer-term data; this should not be interpreted as evidence of durability of effect (see Section 5). (**f**) Reduction in Body Mass Index (BMI) at 12 months: A highly consistent reduction in BMI across all included studies, showing minimal variability in the procedure’s performance. Studies contributing to each panel: (**a**) [31,33,35]; (**b**) [18,28,30,31,32,34,35]; (**c**) [28,30,32,36]; (**d**) [28,30,31,32,34,35]; (**e**) [20,32,34]; (**f**) [28,31,32,35,36].

At 6 months, pooled total body weight loss (%TBWL) was 16.31% (95% CI 15.10 to 17.53; three studies, I^2^ = 0%); applying the prespecified time-window criterion, Abad et al. [17] was excluded from the 12-month analysis because its weight outcome was assessed at week 72 (~16.6 months) and is reported narratively among the randomised between-group findings; at 12 months it was 16.54% (95% CI 14.48 to 18.60), with a mean body-weight change of MD −16.28 kg (95% CI −18.11 to −14.45) and a BMI change of MD −6.21 kg/m^2^ (95% CI −6.31 to −6.11; I^2^ = 0%). Excess weight loss reached 55.73% (95% CI 43.76 to 67.69). At 36 months, %TBWL was 14.21% (95% CI 7.58 to 20.85), suggesting that weight loss remained observable at longer follow-up in some cohorts. However, the 6-, 12-, and 36-month estimates derive from partly non-overlapping sets of studies dominated by different large cohorts (Table 2), so a single-cohort weight-loss trajectory or a peak effect cannot be inferred from these cross-sectional pooled estimates, and the 36-month estimate in particular is based on few studies (Figure 4).

### 4.5. Between-Group Findings from Randomised Trials

Because only two included studies were randomised, between-group effects were not pooled but are summarised here as reported by the original trials. In MERIT [30], ESG plus lifestyle modification was superior to lifestyle modification alone at 52 weeks for glycated haemoglobin (between-group mean difference 0.49%, 95% CI 0.30 to 0.68; *p* < 0.0001), HOMA-IR (2.88, 95% CI 0.99 to 4.77; *p* = 0.0031), fasting glucose (14.42 mg/dL, 95% CI 6.30 to 22.55; *p* = 0.0007), ALT (13.69 U/L, 95% CI 5.75 to 21.63; *p* = 0.0014), AST (5.76 U/L, 95% CI 1.33 to 10.20; *p* = 0.011), and Hepatic Steatosis Index (1.41, 95% CI 0.27 to 2.54; *p* = 0.015), alongside a between-group difference of 12.6% total body weight loss (95% CI 10.7 to 14.5) and 44.7% excess weight loss (95% CI 37.5 to 51.9). In the sham-controlled trial of Abad et al. [17], ESG was superior to sham endoscopy for total body weight loss (9.47% vs. 3.91%; *p* < 0.05), HOMA-IR change (−5.21 vs. −1.98; *p* = 0.013), and liver stiffness by transient elastography (−5.63 kPa vs. −0.2 kPa; *p* = 0.017). Full details are provided in Supplementary Table S2. These randomised, between-group data are consistent in direction with the pooled within-group findings and provide some support that the associations described above are not attributable solely to lifestyle co-intervention, although the small number of randomised trials and outcomes precludes formal pooling or a definitive causal conclusion.

### 4.6. Sensitivity Analyses, Risk of Bias, and Certainty of Evidence

Four sensitivity analyses were performed. First, leave-one-out removal of individual studies did not materially alter the direction of the principal estimates (Supplementary Tables S5 and S6). Second, from Jirapinyo et al. [18], only the endoscopic monotherapy arm was extracted, so no pooled estimate includes patients receiving a GLP-1 receptor agonist; excluding that cohort altogether left the principal estimates essentially unchanged and still significant (glycated haemoglobin MD −0.34, 95% CI −0.51 to −0.17; homeostatic model assessment of insulin resistance MD −3.48, 95% CI −5.01 to −1.95 (three studies, additionally excluding Hajifathalian et al. [19] per the cohort-overlap criterion above); alanine aminotransferase MD −7.78, 95% CI −9.65 to −5.90 (four studies, additionally excluding Espinet-Coll et al. [28] per the data-availability criterion, Abad et al. [17] per the time-window criterion and Hajifathalian et al. [19] per the cohort-overlap criterion above); aspartate aminotransferase was unaffected by this exclusion because Jirapinyo et al. [18] did not contribute to that estimate (MD −2.49, 95% CI −5.50 to 0.53; three studies, which also exclude Abad et al. [17] per the time-window criterion and Lahooti et al. [20], Jagtap et al. [36] and Jirapinyo et al. [18], which did not report post-procedural AST values); NAFLD Fibrosis Score MD −0.57, 95% CI −0.92 to −0.21; %TBWL at 12 months 17.92%, 95% CI 16.22 to 19.62), indicating that this small, highly selected cohort did not drive the pooled estimates. Third, applying the HKSJ adjustment left point estimates identical but widened confidence intervals; most outcomes remained significant, whereas the AST estimate (three studies, after excluding Abad et al. [17] per the time-window criterion and Lahooti et al. [20], Jagtap et al. [36] and Jirapinyo et al. [18], which did not report post-procedural AST) did not reach significance under either model (HKSJ 95% CI −6.32 to 1.35); liver stiffness was not pooled and therefore not subject to this sensitivity analysis. Full HKSJ intervals for all outcomes are provided in Supplementary Table S7. Non-randomised studies were evaluated as having moderate-to-serious risk of bias, and the two randomised trials raised some concerns (Supplementary Figure S2). The overall certainty of evidence was very low for most outcomes and low for BMI, low-density lipoprotein cholesterol and the homeostatic model assessment of insulin resistance (Supplementary Table S8). Fourth, for the seven outcomes reported as separate baseline and follow-up group values (HbA1c, HOMA-IR, triglycerides, LDL and HDL cholesterol, ALT, AST), we re-estimated the standard error using an imputed within-subject correlation of 0.5 to approximate a paired analysis. Point estimates were materially similar, and the direction of every outcome was unchanged; statistical significance was likewise unchanged, except that the aspartate aminotransferase estimate, non-significant under the primary model, became borderline under the paired model (95% CI −4.54 to −0.01). Between-study heterogeneity (I^2^) was generally higher under this model (Supplementary Table S7).

## 5. Discussion

In this updated synthesis, ESG was associated with several improvements in metabolic, hepatic, and anthropometric parameters that accompanied substantial weight loss. As almost all pooled analyses compare within-patient baseline and follow-up values without an external control group, these associations cannot be confidently attributed to ESG itself and may instead reflect concomitant lifestyle modification, pharmacotherapy, regression to the mean, or the natural evolution of metabolic disease over time; this caveat, elaborated further in the Limitations below, applies throughout the interpretation of the findings that follow. Pooled %TBWL was 16.54% at 12 months and 14.21% at 36 months; because these time points are drawn from partly non-overlapping study sets dominated by different large cohorts (Table 2), this pattern should not be read as a within-cohort trajectory implying a peak effect at 12 months, and the more defensible interpretation is that weight loss remained observable at longer follow-up in some cohorts. Prior histologically confirmed cohorts have reported that weight loss exceeding 10% predicts improvement in steatohepatitis and fibrosis [37]; however, this threshold, derived from an external histologically confirmed cohort, should not be read as evidence that our own pooled, non-histological findings reflect a comparable hepatic benefit. The magnitude of a within-group pre-post change does not by itself establish a causal or histological effect, and the wide confidence interval at 36 months further reflects sparse long-term data. These estimates are consistent with the report of Nunes et al. [38], who claimed a mean 17.28% TBWL and a 6.31 kg/m^2^ BMI reduction one year after ESG, and with Marincola et al. [39], who reported excess weight loss of 62.20% for ESG versus 80.32% for LSG. Although bariatric surgery remains the most effective option for durable weight loss, ESG is incisionless, potentially reversible, and has a favourable safety profile, positioning it as an intermediate option between lifestyle intervention and surgery for selected patients [30]. The associations with lipid and glycaemic parameters are concordant with Hanscom et al. [16], who reported comparable reductions in low-density lipoprotein cholesterol, triglycerides, glycated haemoglobin, and insulin resistance. Our work extends that analysis in three respects: it incorporates MASLD-related hepatic outcomes, it adds longer-term (36-month) weight data, and it applies HKSJ inference together with a sensitivity analysis isolating ESG-monotherapy effects. We do not claim a fundamentally different conclusion on the metabolic endpoints despite adding Abad et al. [17] and Jirapinyo et al. [18] as independent new cohorts (Section 1); rather, the present study situates those effects alongside hepatic and durability outcomes within a single framework.

The hepatic findings warrant particular caution. Although alanine aminotransferase and the non-invasive steatosis and fibrosis indices were lower after ESG, these are surrogate rather than histological endpoints, the certainty of evidence was very low, and liver stiffness could be described in only 30 patients from two studies assessed at incompatible follow-up windows and was therefore not pooled. Moreover, the Hepatic Steatosis Index and NAFLD Fibrosis Score are composite indices that incorporate BMI and other metabolic or biochemical variables; their decline after ESG may therefore partly reflect a mathematical consequence of weight loss and metabolic improvement rather than a direct, independent hepatic effect. Throughout this manuscript we describe these findings as changes in liver-related biochemical measures or surrogate indices rather than as reduced hepatic inflammation, improved liver fat, or fibrosis reversal. The single study with paired histology [17] precluded a meta-analytical approach. These data are therefore rather hypothesis-generating and cannot prove or even support regression of necroinflammation or fibrosis. Histology remains the reference standard for staging fibrosis, the principal prognostic determinant in MASLD [40,41].

Pathophysiologically, ESG acts beyond mechanical restriction; delayed gastric emptying, enhanced satiety signalling, and modulation of gut hormones such as GLP-1 may contribute to the metabolic changes observed [12,17], and weight loss may attenuate hepatic lipotoxicity and systemic insulin resistance [42].

Bariatric interventions also modify the gut microbiome in pleotropic ways that may influence host metabolism and inflammation; although direct ESG-specific data are lacking, analogous mechanisms described after bariatric surgery could in part underlie the observed associations [43].

Combining ESG synergistically with emerging pharmacotherapies such as GLP-1 receptor agonists is under active investigation [18]; from that study we extracted only the endoscopic monotherapy arm, so the present estimates describe ESG without concomitant GLP-1 receptor agonist therapy, and excluding that cohort entirely did not materially alter them.

Strengths include the simultaneous appraisal of weight, metabolic, and hepatic outcomes and the larger pooled sample relative to prior syntheses. Limitations are substantial. The evidence base is dominated by small, non-randomised, sometimes retrospective studies, with moderate-to-serious risk of bias and low-to-very-low certainty; most estimates derive from uncontrolled baseline-versus-follow-up comparisons, so the contribution of ESG cannot be separated from that of weight loss, lifestyle modification, or concomitant pharmacotherapy. Regression to the mean, secular trends, and the natural evolution of metabolic and hepatic disease over the observation period cannot be excluded as additional contributors, independent of concomitant lifestyle modification or pharmacotherapy. Dietary or nutritional interventions may independently influence body weight and lipid outcomes and should therefore be considered when interpreting these pre–post changes [44]. Heterogeneity was high for the 12- and 36-month total body weight loss and 12-month excess weight-loss estimates, and for the Hepatic Steatosis Index, the Fibrosis-4 index and triglycerides, but low or absent for the 6-month total body weight loss, body mass index, glycated haemoglobin, insulin resistance, low-density lipoprotein cholesterol and aminotransferase estimates. The included studies differed markedly in study design (two randomised, 10 observational; Table 1), follow-up duration (6 months to 5 years), baseline BMI (32.5–42.7 kg/m^2^), and baseline prevalence of type 2 diabetes (0–83%) and dyslipidaemia (2–69%; Table 1); this clinical heterogeneity probably contributes substantially to the statistical heterogeneity (I^2^) observed for those outcomes. Because the number of studies contributing to each pooled outcome was small (typically 4–8), subgroup analyses by study design or risk-of-bias rating were not statistically feasible and were not performed; this is acknowledged as a limitation, and the observed heterogeneity should be interpreted as reflecting this underlying clinical diversity rather than a single, homogeneous treatment effect. Lifestyle factors (diet, physical activity, alcohol intake) and *Helicobacter pylori* status may influence MetS components and hepatic steatosis and fibrosis, including after bariatric procedures [5] and especially after bariatric surgeries [45]. Lack of standardisation in suturing technique and plication number may further contribute to heterogeneity and affect durability [46,47]. Finally, detailed data on dietary programmes, exercise support, dietitian or multidisciplinary follow-up, adherence, and changes in glucose-lowering, lipid-lowering, or weight-loss medications were not consistently reported across the included studies (Supplementary Table S1), precluding formal analysis of their confounding contribution; these co-interventions remain an important, unmeasured source of heterogeneity that should be considered when interpreting the pooled estimates. Notably, some studies reported only a brief postoperative liquid or texture-modified diet intended for procedural tolerance and safety rather than a sustained dietary intervention [30,35], whereas others described structured, longer-term dietary and exercise support; this distinction should be considered when interpreting pooled estimates as reflecting the effect of ESG rather than co-administered lifestyle intervention. For the weight-loss outcomes specifically, the high heterogeneity (I^2^ up to 100%) likely also reflects variation in baseline BMI across cohorts (32.5–42.7 kg/m^2^), differing follow-up and attrition rates, and a mix of randomised and observational designs with non-standardised ESG technique; because the 6-, 12-, and 36-month pooled estimates are drawn from partly non-overlapping sets of studies (Table 2), they represent heterogeneous cross-sectional summaries rather than a single cohort’s trajectory over time. Approximate 95% prediction intervals are provided in Supplementary Table S7 for the outcomes with sufficient degrees of freedom; they remain wide, underscoring that the effect of ESG on weight loss in a new setting cannot be predicted with precision from the current evidence base. We also re-examined the leave-one-out sensitivity analyses (Supplementary Tables S5 and S6): the original description imprecisely characterised these as removing studies with substantial statistical weight. We therefore independently repeated a complete leave-one-out procedure, sequentially removing each contributing study, for the outcomes for which individual study-level data were available (HbA1c, HOMA-IR, triglycerides, LDL cholesterol, ALT, AST). Most estimates were robust to removal of any single study, but one was not: LDL cholesterol lost significance when Lahooti et al. [20] was excluded (MD −6.21, 95% CI −12.58 to 0.17), illustrating that a complete leave-one-out analysis can reveal fragility that influential-study-only sensitivity checks would miss. AST itself is pooled from only three studies, after excluding Abad et al. [17] (time-window criterion) and Lahooti et al. [20], Jagtap et al. [36] and Jirapinyo et al. [18], which did not report post-procedural AST values; it did not reach statistical significance in the primary analysis (MD −2.49, 95% CI −5.50 to 0.53), now with no detectable heterogeneity (I^2^ = 0%). Complete leave-one-out results for the remaining outcomes (BMI, Hepatic Steatosis Index, NAFLD Fibrosis Score, and 12-month weight change) were also robust to removal of any single study (Supplementary Tables S5 and S6). The AST estimate was non-significant in the primary analysis and remained so throughout the complete leave-one-out procedure; the markedly discordant values previously contributed by Jagtap et al. [36] are no longer included, as that study does not report post-procedural AST (Supplementary Table S5). This additional single-study sensitivity reinforces, rather than changes, the very low-to-low GRADE certainty already assigned to these outcomes (Supplementary Table S8). Finally, only two included studies were randomised (Supplementary Table S2); the remaining, larger body of evidence rests on within-group pre–post comparisons, which are hypothesis-generating and cannot establish causation regardless of the magnitude of the observed change. The included populations were also clinically heterogeneous at baseline: eligibility criteria ranged from a BMI threshold alone to lower BMI thresholds conditional on obesity-related comorbidities, and some studies required biopsy-proven or imaging-confirmed MASLD/MASH for enrolment (Abad et al. [17], Jirapinyo et al. [18], Hajifathalian et al. [19], Jagtap et al. [36], Espinet-Coll et al. [28]), while others enrolled unselected patients with obesity in whom liver disease was assessed only as an outcome (Supplementary Table S1). Consequently, the pooled hepatic estimates combine cohorts pre-selected for MASLD with cohorts of unknown baseline liver status, which likely contributes to the observed heterogeneity and means the pooled effect should not be interpreted as applying uniformly to a single, well-defined MASLD population. Regarding MetS definitions specifically, none of the included studies required a formal composite metabolic syndrome diagnosis for eligibility (Supplementary Table S1), so differing diagnostic frameworks (ATP III, AHA/NHLBI, or IDF) are unlikely to have influenced study selection; however, this same definitional inconsistency across studies is precisely why complete metabolic syndrome resolution could not be reliably pooled as originally intended (Section 2), motivating the post hoc focus on individual, directly extracted components (glycaemic status, lipid fractions, blood pressure), which are not dependent on any single composite definition.

In conclusion, using a post hoc analysis of individual MetS components in place of the unpooled composite outcome, and in a predominantly observational, low-certainty evidence base, ESG was associated with improvements in glycaemic, lipid, and liver-related parameters alongside substantial and partly sustained weight loss. These associations are promising but hypothesis-generating and cannot establish a weight-independent effect of ESG. Adequately powered randomised trials with paired liver biopsies are necessitated to determine whether ESG affects MetS and MASLD beyond weight reduction.

## 6. Summary Box

*What is already known:*

- Endoscopic sleeve gastroplasty produces clinically meaningful weight loss with a favourable safety profile relative to bariatric surgery.
- Prior meta-analyses have addressed either metabolic syndrome components or MASLD-related hepatic outcomes separately, in smaller datasets limited to 12 months; none has integrated both domains together with longer-term weight data.
- Weight loss exceeding 10% predicts histological improvement in steatohepatitis and fibrosis.
- The certainty of evidence for endoscopic sleeve gastroplasty across metabolic and hepatic outcomes has not been formally graded.

*What are the new findings:*

- Endoscopic sleeve gastroplasty was associated with improvements in glycaemic, lipid, and hepatic parameters alongside 17% total body weight loss at 12 months, partly sustained to 36 months.
- The core metabolic and weight findings were robust to exclusion of the single cohort drawn from a combination-therapy study and to conservative Hartung–Knapp–Sidik–Jonkman sensitivity analyses, and, with the single exception of low-density lipoprotein cholesterol, to complete leave-one-out removal; liver stiffness was reported by only two studies (30 patients) at incompatible follow-up windows and was therefore not pooled.
- The liver-related evidence rests largely on surrogate markers and small samples and should be regarded as exploratory; HSI and NFS are composite indices that partly reflect BMI and metabolic variables rather than direct hepatic effects.
- The overall certainty of evidence was graded low to very low, warranting therefore randomised trials with histological endpoints.

## Figures and Tables

**Table 1:** Baseline demographic and clinical characteristics of patients undergoing ESG in the included studies.

1st Author, Year	Country	Design	Sample Size (ESG)	Mean Age (Years, ±SD)	Males	Follow-Up (Months)	Baseline BMI (kg/m^2^, Mean, ±SD)	Baseline Mean Weight (kg, ±SD)	Baseline Mean Waist (cm, ±SD)	Baseline AH (n, %)	Baseline Dyslipidemia (n, %)	Baseline T2DM (n,%)
Abad 2025 [17]	Spain	RCT	20	55.15 (10.9)	11	11/17 ^b^	37.54 (4.81)	106.15 (21.85)	118.72 (13.12)	11 (55)	12 (69)	9 (45)
Abu Dayyeh 2022 [30]	USA	RCT	77	47.30 (9.30)	9	12	35.5 (2.6)	98.4 (12.3)	110.3 (10.4)	38 (49)	13 (17)	18 (23)
Alexandre 2023 [31]	France	Retrospective	99	45 (12.70)	25	6, 12	42.7 (7.8)	119.8 (24.1)	NA	38 (38.4)	26 (26.3)	26 (26.3)
Alqahtani 2022 [32]	Saudi Arabia	Retrospective/Registry	3018	33.8 (9.6)	332	6 to 36	32.5 (3.1)	NA	NA	101 (3.3)	62 (2.1)	112 (3.7)
Asokkumar 2021 [33]	Singapore	Retrospective	35	43.60 (11.30)	15	3 and 6	34 (4.9)	93.2 (16)	NA	17 (49)	NA	8 (23)
Bhandari 2023 [34]	India	Prospective	612	40.7 (12.65)	188	48	34.3 (5.05)	90.37 (16.61)	NA	366 (59.8)	410 (67)	271 (44.2)
Carr 2022 [35]	Australia	Two arm Prospective	16	41.40 (10.40)	3	6, 12	35.5 (5.2)	NA	NA	7 (44)	3 (19)	0
Espinet-Coll 2019 [28]	Spain	Two arm Prospective	15	46.90 (15.50)	4	12	39.82 (6.78)	107.89 (19.1)	121 (13.1)	5 (33)	8 (53.3)	3 (20)
Hajifathalian 2021 [19] ^c^	USA	Prospective	118	46 (13)	38	12, 24	40 (7)	NA	NA	NA	NA	35 (30)
Jagtap 2021 [36]	India	Prospective	26	41.50 (9.58)	10	12	36.55 (5.07)	99.43 (21.89)	NA	19 (73.1)	17 (65.4)	13 (50)
Jirapinyo 2024 [18]	USA	Retrospective	12	51 (14)	3	12	38 (6.7)	NA	NA	NA	NA	10 (83)
Lahooti 2025 [20] ^c^	USA	Prospective	404	45 (11.9)	97	12, 36, 60	37.3 (5.8)	NA	NA	234 (57.9)	260 (64.4)	57 (14.1)

Abbreviations: AH, arterial hypertension; BMI, body mass index; ESG, endoscopic sleeve gastroplasty; kg, kilograms; m^2^, square meters; NA, not available; n, number of patients; RCT, randomised controlled trial; SD, standard deviation; T2DM, type 2 diabetes mellitus. ^b^ Abad et al. [17] assessed outcomes at multiple visits (weeks 12, 24, 48, and 72); HOMA-IR was assessed at week 48 (~11.1 months, within the prespecified 9–15 month window and retained in the 12-month analysis), whereas ALT, AST, liver stiffness, and total body weight loss were assessed at week 72 (~16.6 months) and were therefore excluded from the 12-month pooled analyses and reported narratively (see Section 2, Table 2, and Section 4). ^c^ Hajifathalian et al. [19] and Lahooti et al. [20] originate from the same institution and senior author, with overlapping recruitment windows; Hajifathalian et al. [19] was excluded from the HOMA-IR and ALT pooled analyses to avoid double-counting of overlapping participants with Lahooti et al. [20] (see Section 2).

**Table 2:** Outcomes included in the quantitative synthesis and data availability.

Category	Outcome	Time Point	No. of Studies	Patients (n)	Paired/Follow-Up N	Attrition to Follow-Up (%)
Weight loss	%TBWL	6 months	3	150	NR	NR
	%TBWL	12 months	7	3849	NR	NR
	%TBWL	36 months	3	4034	NR	NR
	%EWL	12 months	6	3837	NR	NR
	Body weight (kg)	12 months	4	3136	NR	NR
	BMI (kg/m^2^)	12 months	5	3174	NR	NR
Metabolic	HbA1c	12 months	6	537	436	18.8%
	HOMA-IR	12 months	4 ^c^	451	381	15.5%
	Triglycerides	12 months	5	611	510	16.5%
	LDL cholesterol	12 months	5	608	511	16.0%
	HDL cholesterol	12 months	4	204	175	14.2%
	ALT	12 months	5 ^bce^	557	478	14.2%
	AST	12 months	3 ^bd^	130	119	8.5%
	Hepatic Steatosis Index	12 months	5	252	NR	NR
	NAFLD Fibrosis Score	12 months	4	171	NR	NR
	FIB-4	12 months	3	53	NR	NR
	Liver stiffness (kPa) ^ab^	—	not pooled (k = 2)	30	not pooled	not pooled

Counts reflect the studies that contributed usable quantitative data (mean and standard deviation or standard error) to each pooled analysis at the specified time point; patient numbers are the baseline endoscopic sleeve gastroplasty cohorts entering each analysis. For outcomes reported by primary studies as separate baseline and follow-up group values (HbA1c, HOMA-IR, triglycerides, LDL and HDL cholesterol, ALT, AST), the “Paired/follow-up N” column reports the number of participants with a follow-up measurement at the specified time point, and attrition is the proportion of the baseline cohort without follow-up data; for outcomes reported only as a within-subject mean change (weight-loss metrics, Hepatic Steatosis Index, NAFLD Fibrosis Score, FIB-4, liver stiffness), primary studies did not separately report baseline and follow-up sample sizes, so paired attrition could not be independently determined from the published aggregate data and is marked not reported (NR); the “Patients (n)” column reports the baseline endoscopic sleeve gastroplasty cohort entering the analysis, as it does for every other outcome, and should not be read as an independently verified paired sample. ^a^ The liver stiffness measurement analysis is based on only two studies (30 patients) and is reported as exploratory. Categorical comorbidity outcomes (arterial hypertension, type 2 diabetes mellitus, dyslipidaemia) were reported descriptively by individual studies and were not pooled. ALT, alanine aminotransferase; AST, aspartate aminotransferase; BMI, body mass index; %EWL, percentage excess weight loss; FIB-4, fibrosis-4 index; HbA1c, glycated haemoglobin; HDL, high-density lipoprotein; HOMA-IR, homeostatic model assessment of insulin resistance; LDL, low-density lipoprotein; NAFLD, non-alcoholic fatty liver disease; %TBWL, percentage total body weight loss. ^b^ Following the prespecified time-window criterion (Section 2; 12-month bucket = 9–15 months), the sham-controlled trial of Abad et al. [17] was excluded from the ALT, AST and 12-month %TBWL pooled analyses (these outcomes assessed at week 72, ~16.6 months) and is reported narratively; liver stiffness (only two studies, at 12 months and week 72 respectively) was not pooled for the same reason and is also reported narratively in the Section 4. ^c^ Hajifathalian et al. [19] was additionally excluded from the HOMA-IR and ALT pooled analyses because of its cohort overlap with Lahooti et al. [20] (same institution and senior author, overlapping recruitment windows); see Section 2 and Table 1. ^d^ Lahooti et al. [20], Jirapinyo et al. [18] and Jagtap et al. [36] did not report post-procedural AST values in the original publications and were therefore excluded from the AST pooled analysis (Supplementary Table S4). ^e^ Espinet-Coll et al. [28] reported ALT as a median with interquartile range and, in the ESG column, printed values identical to those of the adjacent gamma-glutamyl transferase row, so no usable mean and standard deviation could be recovered; that study was therefore excluded from the ALT pooled analysis. Its triglyceride values were likewise reported as a median with interquartile range, but with identical medians at both timepoints, indicating a symmetrical distribution, and were therefore treated as a mean with standard deviation; this is conservative because the interquartile range exceeds the corresponding standard deviation and so reduces rather than increases that study’s weight.

## Data Availability

All data analysed are derived from previously published studies cited in the manuscript; all extracted data are available within the article and its Supplementary Materials. The review protocol is registered in PROSPERO (CRD420261283041).

## Supplementary Materials

The following supporting information can be downloaded at https://www.mdpi.com/article/10.3390/jcm15176633/s1, Figure S1: funnel plot for the best-populated outcome; Figure S2: risk-of-bias assessment (ROBINS-I and RoB 2); Table S1: study eligibility criteria, liver-disease definitions and co-interventions; Table S2: between-group findings from the two randomised trials; Table S3: detailed search strategy; Table S4: baseline laboratory values; Table S5: complete leave-one-out sensitivity analysis (part 1 of 2); Table S6: complete leave-one-out sensitivity analysis (part 2 of 2); Table S7: DerSimonian–Laird versus Hartung–Knapp–Sidik–Jonkman sensitivity estimates; Table S8: GRADE summary of findings.

## Abbreviations

AH	Arterial Hypertension
ALT	Alanine Aminotransferase
AST	Aspartate Aminotransferase
BMI	Body Mass Index
CAP	Controlled Attenuation Parameter
CI	Confidence Interval
ESG	Endoscopic Sleeve Gastroplasty
EWL	Excess Weight Loss
FIB-4	Fibrosis-4 Index
HbA1c	Glycated Haemoglobin
HDL	High-Density Lipoprotein
HKS	Hartung–Knapp–Sidik–Jonkman
HOMA-IR	Homeostatic Model Assessment of Insulin Resistance
HSI	Hepatic Steatosis Index
IR	Insulin Resistance
LDL	Low-Density Lipoprotein
LSG	Laparoscopic Sleeve Gastrectomy
LSM	Liver Stiffness Measurement
MASLD	Metabolic Dysfunction-Associated Steatotic Liver Disease
MASH	Metabolic Dysfunction-Associated Steatohepatitis
MD	Mean Difference
MetS	Metabolic Syndrome
MRE	Magnetic Resonance Elastography
MRI-PDFF	Magnetic Resonance Imaging–Proton Density Fat Fraction
NAFLD	Non-Alcoholic Fatty Liver Disease
NASH	Non-Alcoholic Steatohepatitis
NFS	NAFLD Fibrosis Score
PICO	Population, Intervention, Comparison, Outcome
PRISMA	Preferred Reporting Items for Systematic Reviews and Meta-Analyses
RCT	Randomised Controlled Trial
ROBINS-I	Risk Of Bias In Non-randomised Studies of Interventions
RoB 2	Risk of Bias Tool version 2
SD	Standard Deviation
SE	Standard Error
TBWL	Total Body Weight Loss
TG	Triglycerides
T2DM	Type 2 Diabetes Mellitus
VCTE	Vibration-Controlled Transient Elastography
WHO	World Health Organization
