# Supplementary material for: Beyond Weight Reduction: An Updated Systematic Review and Meta-Analysis of Metabolic Syndrome Components and Metabolic Dysfunction-Associated Steatotic Liver Disease Outcomes After Endoscopic Sleeve Gastroplasty
Source: J Clin Med. 2026 Aug 27;15(17):6633. doi: 10.3390/jcm15176633 (PMC13566304; doi:10.3390/jcm15176633)
Supplement: Supplementary file 1 [file jcm-15-06633-s001.zip › Supplementary Table S8.pdf]

Supplementary Table S8. GRADE summary of findings: certainty of the evidence for the pooled outcomes after endoscopic sleeve gastroplasty.

Certainty assessment							№ of patients		Effect		Certainty	Importance
№ of studies	Study design	Risk of bias	Inconsistency	Indirectness	Imprecision	Other considerations	Baseline	Follow-up (paired)	Relative (95% CI)	Absolute (95% CI)		
**HbA1c (%) baseline vs 12 months**												
6	non-randomised studies	serious[a]	not serious	not serious	serious[b]	none	537	436	-	MD **0.33 lower** (0.18 lower to 0.47 lower)	⊕◯◯◯ Very low [a,b]	IMPORTANT
**ALT (U/L) baseline vs 12 months post ESG**												
5	non-randomised studies	serious[a]	not serious	not serious	not serious	none	557	478	-	MD **7.82 lower** (5.95 lower to 9.69 lower)	⊕◯◯◯ Very low [a]	IMPORTANT
**HOMA-IR baseline vs 12 months post ESG**												
4	non-randomised studies	serious[a]	not serious	not serious	not serious	strong association	451	381	-	MD **3.52 lower** (2.18 lower to 4.87 lower)	⊕⊕◯◯ Low [a]	IMPORTANT
**NFS**												
4	non-randomised studies	serious[a]	not serious	not serious	serious[b]	none	171	NR	-	MD **0.54 lower** (0.26 lower to 0.82 lower)	⊕◯◯◯ Very low [a,b]	CRITICAL
**LDL**												
5	non-randomised studies	serious[a]	not serious	not serious	not serious	strong association	608	511	-	MD **6.94 lower** (3.65 lower to 10.22 lower)	⊕⊕◯◯ Low [a]	IMPORTANT
**BMI**												

Certainty assessment							№ of patients		Effect		Certainty	Importance
№ of studies	Study design	Risk of bias	Inconsistency	Indirectness	Imprecision	Other considerations	Baseline	Follow-up (paired)	Relative (95% CI)	Absolute (95% CI)		
5	non-randomised studies	serious[a]	not serious	not serious	not serious	strong association	3174	NR	-	MD **6.21 lower** (6.11 lower to 6.31 lower)	⨁⨁◯◯ Low[a]	CRITICAL
**%TBWL 12m**												
7	non-randomised studies	serious[a]	very serious[c]	not serious	not serious	strong association	3849	NR	-	MD **16.54 lower** (14.48 lower to 18.60 lower)	⨁◯◯◯ Very low[a,c]	CRITICAL

CI: confidence interval; **MD:** mean difference

Explanations

a. downgraded due to non-randomized design

b. downgraded due to small sample size

c. downgraded due to severe heterogeneity (I^2>75%)
