# Supplementary material for: Beyond Weight Reduction: An Updated Systematic Review and Meta-Analysis of Metabolic Syndrome Components and Metabolic Dysfunction-Associated Steatotic Liver Disease Outcomes After Endoscopic Sleeve Gastroplasty
Source: J Clin Med. 2026 Aug 27;15(17):6633. doi: 10.3390/jcm15176633 (PMC13566304; doi:10.3390/jcm15176633)
Supplement: Supplementary file 1 [file jcm-15-06633-s001.zip › Supplementary Table S1.pdf]

Supplementary Table S1. Key eligibility criteria, liver-disease definitions, and reported co-interventions of the included studies

1st author, year	Original BMI eligibility	Metabolic comorbidities – eligibility	Liver disease definition	Previous conservative weight-management failure	Key exclusions	Dietary & exercise program	Dietitian / multidisciplinary follow-up	Adherence assessment reported	Medication changes tracked (glucose-/lipid-lowering, weight-loss)
Abad 2025 [17]	BMI ≥30 kg/m²	No separate metabolic comorbidity requirement; MASH itself required	Biopsy-proven MASH, NAS ≥3 with ≥1 point each for lobular inflammation and ballooning; fibrosis F0–F3	NR	Other causes of liver disease; abusive alcohol consumption; cirrhosis/advanced fibrosis beyond F3	Structured lifestyle modification in both arms (ESG+LM vs sham+LM); Mediterranean-diet counselling	NR (not itemised)	Yes – Mediterranean-diet adherence (PREDIMED) and physical activity (IPAQ) compared between arms	NR
Abu Dayyeh 2022 [29]	BMI 30–40 kg/m²	No individual comorbidity required; prespecified recruitment quotas for HTN and T2DM, with ≤50 participants without comorbidity	No liver disease required; hepatic insufficiency/cirrhosis excluded	Yes; failure of nonsurgical weight-loss methods	Prior foregut/GI or bariatric surgery; significant GI inflammatory/structural disease; severe coagulopathy; insulin-dependent diabetes; hepatic insufficiency/cirrhosis; active psychological disease; selected medications and major respiratory disease	Individualised low-calorie diet plan + physical-activity counselling, reiterated at each visit; lifelong dietary restriction required	Yes – lifestyle counselling at each study visit	Yes – lifestyle-modification adherence incentivised/monitored; crossover eligibility tied to visit attendance	Yes – antihypertensive, antidiabetic and lipid-lowering medication status tracked
Alexandre 2023 [30]	BMI ≥30 kg/m² with comorbidities	Yes – obesity with comorbidities required, but no	No specific liver disease requirement	Yes; failure of medical treatment	Esophageal pathology; previous bariatric/gastric/esophageal surgery;	Protocol-prescribed diet/lifestyle	Scheduled consultations incl. anesthesiologis	Non-adherence noted as cause of dropout; not	Yes – antihypertensive/ antidiabetic medication status

1st author, year	Original BMI eligibility	Metabolic comorbidities – eligibility	Liver disease definition	Previous conservative weight-management failure	Key exclusions	Dietary & exercise program	Dietitian / multidisciplinary follow-up	Adherence assessment reported	Medication changes tracked (glucose-/lipid-lowering, weight-loss)
		specific metabolic comorbidity mandated			esophageal varices; severe coagulopathy; unstable heart disease; uncontrolled eating disorder	measures (details NR)	ts and other specialists (multidisciplinary bariatric preparation programme); full team composition not itemised	formally quantified	used to define comorbidities
Alqahtani 2022 [31]	BMI 27.5–35 kg/m²; also patients eligible for bariatric surgery who declined surgery regardless of BMI	No specific metabolic comorbidity requirement	No specific liver disease requirement	NR; lifestyle/nutritional counselling was provided before intervention	Specific exclusion criteria NR in the published Methods; prior bariatric/gastric/esophageal surgery and other procedural contraindications were generally considered in practice	Individualised management plan incl. nutritional counselling and behavioural therapy; exercise NR	Yes – multidisciplinary team, monthly online visits throughout follow-up	NR	Yes – antihypertensive/ antidiabetic medication status used to define comorbidity remission
Asokkumar 2021 [32]	BMI ≥27.5 kg/m²	No specific metabolic comorbidity required	No specific liver disease requirement; fatty liver and cirrhosis were baseline comorbidities	Yes; failed diet and lifestyle therapy; all had declined surgery	Severe systemic illness; substance abuse; uncontrolled eating disorder; pregnancy; coagulopathy	Structured post-op diet progression (4-wk liquid→solid) with Harris–Benedict energy	Yes – regular follow-up by dietitians, gastroenterologists, physiotherapists	NR for diet/lifestyle (only procedural suturing adherence reported)	Yes – antidiabetic/antihypertensive medication reduction or discontinuation tracked

1st author, year	Original BMI eligibility	Metabolic comorbidities – eligibility	Liver disease definition	Previous conservative weight-management failure	Key exclusions	Dietary & exercise program	Dietitian / multidisciplinary follow-up	Adherence assessment reported	Medication changes tracked (glucose-/lipid-lowering, weight-loss)
						prescription; individualised exercise plan			
Bhandari 2023 [33]	BMI >30 kg/m², or >27 kg/m² with comorbidities	No specific metabolic comorbidity required	No specific liver disease requirement	NR	NR	Post-op diet progression only (liquid→modified bariatric diet); exercise NR	Multidisciplinary care described generally (nutrition, psychiatry, endocrinology); not itemised per patient	NR	Pharmacotherapy use mentioned qualitatively; not systematically tracked
Carr 2022 [34]	BMI >30 kg/m² (obese category); or BMI >26 kg/m² with comorbidities (hypertension, T2DM, or cardiovascular disease) per site proceduralist eligibility criteria (Carr et al. [34], Suppl.	No specific metabolic comorbidity required for the BMI >30 kg/m² pathway; hypertension, T2DM, or cardiovascular disease permitted the BMI >26 kg/m² pathway (Carr	No specific liver disease diagnosis required for inclusion; hepatic biochemistry (HSI) assessed as an outcome. Liver cirrhosis (particularly with portal hypertension, coagulopathy, or thrombocytopenia) was a	NR	Residence >1.5 h from study site; per Carr et al. [34], Suppl. Table S1 (proceduralist contraindications): liver cirrhosis (esp. with portal hypertension, coagulopathy, or thrombocytopenia), medical comorbidity precluding general anesthesia, current/planned	Yes – VLCD pre-op (1–3 wk by BMI), 6-wk texture-modified diet post-op, dietitian-directed; exercise discussed as a recommendation, not a prescribed protocol	Yes – 12-month standard multidisciplinary follow-up (dietitian, nurse, psychologist; Carr et al. [34], Suppl. Table S2)	Dietary advice aimed at "adherence"; not quantified	NR

1st author, year	Original BMI eligibility	Metabolic comorbidities – eligibility	Liver disease definition	Previous conservative weight-management failure	Key exclusions	Dietary & exercise program	Dietitian / multidisciplinary follow-up	Adherence assessment reported	Medication changes tracked (glucose-/lipid-lowering, weight-loss)
	Table S1) – no fixed numeric threshold stated in the primary study protocol	et al. [34], Suppl. Table S1)	contraindication (Carr et al. [34], Suppl. Table S1)		pregnancy (12–18 months), psychiatric disorders affecting weight loss or safety, anatomic variation precluding ESG, mucosal bleeding lesions, neoplastic/premalignant lesions, large hiatal hernia (>3 cm), portal hypertension (varices/gastropathy)				
Espinet-Coll 2019 [35]	BMI 30–40 kg/m²; grade III obesity if surgery refused or patient unsuitable for surgery	No separate metabolic comorbidity requirement	NAFLD defined by any degree of ultrasound hepatic steatosis	Yes; ≥1 ineffective controlled dietary intervention or previous weight regain	Usual contraindications to bariatric endoscopy; viral/autoimmune/metabolic liver disease excluded; significant alcohol intake excluded	Yes – staged diet (liquid→soft→ Mediterranean, fructose-restricted) controlled as the only modified variable; exercise NR	Yes – nutritionist/multidisciplinary review	NR	Yes – antidiabetic drug discontinuation tracked in T2DM patients
Hajifathalian 2021 [19]	BMI >30 kg/m²; BMI >40 kg/m² allowed if surgery refused	No specific metabolic comorbidity required	NAFLD (required for inclusion) identified by HSI	Yes; failure of previous noninvasive weight-loss	Previous bariatric procedures; family history of gastric cancer; neoplastic	Full liquid diet for first 2 weeks post-op, then dietitian-	Yes – multidisciplinary evaluation pre-ESG	NR	Failure of prior pharmacotherapy was an eligibility criterion; post-

1st author, year	Original BMI eligibility	Metabolic comorbidities – eligibility	Liver disease definition	Previous conservative weight-management failure	Key exclusions	Dietary & exercise program	Dietitian / multidisciplinary follow-up	Adherence assessment reported	Medication changes tracked (glucose-/lipid-lowering, weight-loss)
	or patient not a surgical candidate		>36; fibrosis risk estimated by NFS	measures, including pharmacotherapy, to achieve sustainable ≥5% TBWL	gastric lesions; significant mental health disorders; coagulopathy; comorbidities precluding deep sedation	directed diet continuation; exercise NR	(gastroenterologist, endocrinologist, dietitian, psychologist) and dietitian follow-up		ESG medication changes NR
Jagtap 2021 [36]	BMI >27.5 kg/m²	No separate metabolic comorbidity requirement	Fatty liver with altered LFTs, ultrasound evidence of steatosis and HSI >36	Yes; failed lifestyle intervention ± pharmacotherapy	Active gastroduodenal ulcer; large hiatal hernia; varices/coagulopathy; family history of gastric carcinoma; significant alcohol intake; hepatitis B/C; medications inducing fatty liver	Yes – supervised post-op diet progression (liquid→regular, 3–4 wk); exercise NR	Yes – multidisciplinary team (endocrinologist, hepatologist, nutritionist, physiotherapist, psychologist)	NR	Yes – antidiabetic medication/insulin changes reported
Jirapinyo 2024 [18]	BMI ≥30 kg/m²	MASLD required; no separate diabetes/HTN requirement	MASLD: hepatic steatosis on imaging or histology without other identifiable causes; cACLD: F 3 or above, with LSM ≥8 kPa by VCTE	NR	Non-GLP-1RA AOM use or GLP-1RA initiated outside the prespecified 6-month window	Post-op liquid diet for 6 weeks (institutional protocol); no structured long-term programme reported	NR	NR	Yes (study focus) – GLP-1RA type/timing explicitly tracked relative to EGR

1st author, year	Original BMI eligibility	Metabolic comorbidities – eligibility	Liver disease definition	Previous conservative weight-management failure	Key exclusions	Dietary & exercise program	Dietitian / multidisciplinary follow-up	Adherence assessment reported	Medication changes tracked (glucose-/lipid-lowering, weight-loss)
Lahooti 2025 [20]	BMI ≥30 kg/m², or ≥27 kg/m² with comorbidities	No specific metabolic phenotype required; comorbidities permitted the BMI ≥27 pathway	No specific liver disease required; MASLD and liver biomarkers assessed as outcomes	Yes; failed prior nonsurgical weight-loss attempts or, if surgery-eligible, chose not to undergo surgery	Previous Roux-en-Y gastric bypass; pregnancy; uncontrolled psychiatric illness; personal history of gastric cancer; unstable weight-loss medication dosing	Structured post-op nutrition follow-up: bimonthly visits for first 3 months, then monthly	Yes – dietitian, psychologist, and endocrinologist (when indicated) evaluation	NR	Yes – baseline weight-loss medication dose stability required; antihypertensive medication use tracked; post-ESG adjuvant AOM initiation recorded but such patients excluded from final comorbidity analysis (used only to censor pre-medication weight)

Abbreviations: BMI, body mass index; cACLD, compensated advanced chronic liver disease; EGR, endoscopic gastric remodeling; ESG, endoscopic sleeve gastroplasty; GLP-1RA, glucagon-like peptide-1 receptor agonist; HSI, hepatic steatosis index; HTN, hypertension; LSM, liver stiffness measurement; MASLD, metabolic dysfunction-associated steatotic liver disease; MASH, metabolic dysfunction-associated steatohepatitis; NAFLD, nonalcoholic fatty liver disease; NAS, nonalcoholic fatty liver disease activity score; NFS, NAFLD fibrosis score; NR, not reported; T2DM, type 2 diabetes mellitus; TBWL, total body weight loss; VCTE, vibration-controlled transient elastography.
