# Supplementary material for: Beyond Weight Reduction: An Updated Systematic Review and Meta-Analysis of Metabolic Syndrome Components and Metabolic Dysfunction-Associated Steatotic Liver Disease Outcomes After Endoscopic Sleeve Gastroplasty
Source: J Clin Med. 2026 Aug 27;15(17):6633. doi: 10.3390/jcm15176633 (PMC13566304; doi:10.3390/jcm15176633)
Supplement: Supplementary file 1 [file jcm-15-06633-s001.zip › Supplementary Table S2.pdf]

Supplementary Table S2. Between-group findings from the two randomised trials (not pooled).

Trial	Comparison	Outcome	ESG arm	Control arm	Between-group difference (95% CI)	P-value
MERIT (Abu Dayyeh et al., 2022) [29]	ESG+lifestyle vs lifestyle alone, 52 weeks	%TBWL	—	—	12.6% (10.7 to 14.5)	<0.0001
MERIT	ESG+lifestyle vs lifestyle alone, 52 weeks	%EWL	—	—	44.7% (37.5 to 51.9)	<0.0001
MERIT	ESG+lifestyle vs lifestyle alone, 52 weeks	HbA1c (%)	-0.36 (0.665)	0.13 (0.456)	0.49 (0.30 to 0.68)	<0.0001
MERIT	ESG+lifestyle vs lifestyle alone, 52 weeks	HOMA-IR	-1.44 (4.313)	1.44 (5.933)	2.88 (0.99 to 4.77)	0.0031
MERIT	ESG+lifestyle vs lifestyle alone, 52 weeks	Fasting glucose (mg/dL)	-6.11 (23.818)	8.32 (23.509)	14.42 (6.30 to 22.55)	0.0007
MERIT	ESG+lifestyle vs lifestyle alone, 52 weeks	ALT (U/L)	-13.47 (26.328)	0.22 (20.477)	13.69 (5.75 to 21.63)	0.0014
MERIT	ESG+lifestyle vs lifestyle alone, 52 weeks	AST (U/L)	-4.82 (12.223)	0.94 (13.374)	5.76 (1.33 to 10.20)	0.011
MERIT	ESG+lifestyle vs lifestyle alone, 52 weeks	Hepatic Steatosis Index	-2.02 (3.15)	-0.61 (3.409)	1.41 (0.27 to 2.54)	0.015
Abad et al., 2025 [17]	ESG vs sham endoscopy, both + lifestyle, 48–72 weeks	%TBWL	9.47 (9.38)	3.91 (5.43)	not reported (P<0.05)	<0.05
Abad et al., 2025 [17]	ESG vs sham endoscopy, 48 weeks	HOMA-IR (change)	-5.21 (4.93)	-1.98 (8.45)	not reported	0.013
Abad et al., 2025 [17]	ESG vs sham endoscopy, 72 weeks	Liver stiffness, transient elastography	-5.63 (7.2)	-0.2 (5.38)	not reported	0.017

(kPa)

Values are mean (SD) unless otherwise stated. Data are as reported in the original trial publications and supplementary appendices (MERIT: Abu Dayyeh et al. [29], Lancet 2022, Table S9; Abad et al. [17], Clin Gastroenterol Hepatol 2025). Between-group differences for MERIT metabolic and hepatic outcomes are model-adjusted estimates from the original trial (Table S9 of the MERIT supplementary appendix); between-group differences for Abad et al. were reported as P-values for group comparison without a published effect estimate and 95% CI, and are indicated as "not reported" here. These results are presented narratively and were not included in the pooled random-effects meta-analysis, given the small number of randomised trials and the heterogeneity of reported outcomes and comparators (lifestyle-only vs sham-endoscopy control). ALT, alanine aminotransferase; AST, aspartate aminotransferase; %EWL, percentage excess weight loss; HbA1c, glycated haemoglobin; HOMA-IR, homeostatic model assessment of insulin resistance; %TBWL, percentage total body weight loss.
