# Supplementary material for: Beyond Weight Reduction: An Updated Systematic Review and Meta-Analysis of Metabolic Syndrome Components and Metabolic Dysfunction-Associated Steatotic Liver Disease Outcomes After Endoscopic Sleeve Gastroplasty
Source: J Clin Med. 2026 Aug 27;15(17):6633. doi: 10.3390/jcm15176633 (PMC13566304; doi:10.3390/jcm15176633)
Supplement: Supplementary file 1 [file jcm-15-06633-s001.zip › Supplementary Table S3.pdf]

Supplementary Table S3. Detailed search strategy and results

Database	Search Strategy (exact string)	Date of Search	Records Retrieved (n)
**PubMed (MEDLINE)**	(“endoscopic sleeve gastroplasty”[Title/Abstract] OR ESG[Title/Abstract] OR “endoscopic gastric remodeling”[Title/Abstract] OR “endoscopic gastroplasty”[Title/Abstract] OR “bariatric endoscopy”[Title/Abstract] OR “endoscopic bariatric therapy”[Title/Abstract] OR “Apollo OverStitch”[Title/Abstract] OR OverStitch[Title/Abstract]) AND (“metabolic syndrome”[Title/Abstract] OR “metabolic dysfunction”[Title/Abstract] OR “nonalcoholic fatty liver disease”[Title/Abstract] OR “non-alcoholic fatty liver disease”[Title/Abstract] OR NAFLD[Title/Abstract] OR MASLD[Title/Abstract] OR MASH[Title/Abstract] OR NASH[Title/Abstract] OR “hepatic steatosis”[Title/Abstract] OR “fatty liver”[Title/Abstract] OR “steatohepatitis”[Title/Abstract] OR “insulin resistance”[Title/Abstract] OR “HbA1c”[Title/Abstract] OR “HOMA-IR”[Title/Abstract] OR “FIB-4”[Title/Abstract] OR NFS[Title/Abstract] OR CAP[Title/Abstract] OR “controlled attenuation parameter”[Title/Abstract] OR “MRI-PDFF”[Title/Abstract] OR “liver stiffness”[Title/Abstract] OR elastography[Title/Abstract] OR “transient elastography”[Title/Abstract] OR “shear wave elastography”[Title/Abstract] OR FibroScan[Title/Abstract] OR VCTE[Title/Abstract] OR “MR elastography”[Title/Abstract] OR MRE[Title/Abstract] OR biopsy[Title/Abstract] OR histologic[Title/Abstract] OR histological[Title/Abstract] OR histopathologic[Title/Abstract] OR histopathological[Title/Abstract] OR fibrosis[Title/Abstract])	8 Nov 2025	**95**
**Embase (Elsevier)**	(‘endoscopic sleeve gastroplasty’:ab,ti OR ESG:ab,ti OR ‘endoscopic gastric remodeling’:ab,ti OR ‘endoscopic gastroplasty’:ab,ti OR ‘bariatric endoscopy’:ab,ti OR ‘endoscopic bariatric therapy’:ab,ti OR ‘Apollo OverStitch’:ab,ti OR OverStitch:ab,ti) AND (‘metabolic syndrome’:ab,ti OR ‘metabolic dysfunction’:ab,ti OR ‘nonalcoholic fatty liver disease’:ab,ti OR ‘non-alcoholic fatty liver disease’:ab,ti OR NAFLD:ab,ti OR MASLD:ab,ti OR MASH:ab,ti OR NASH:ab,ti OR ‘hepatic steatosis’:ab,ti OR ‘fatty liver’:ab,ti OR ‘steatohepatitis’:ab,ti OR ‘insulin resistance’:ab,ti OR HbA1c:ab,ti OR ‘HOMA-IR’:ab,ti OR ‘FIB-4’:ab,ti OR NFS:ab,ti OR CAP:ab,ti OR ‘controlled attenuation parameter’:ab,ti OR ‘MRI-PDFF’:ab,ti OR ‘liver stiffness’:ab,ti OR elastography:ab,ti OR ‘transient elastography’:ab,ti OR ‘shear wave elastography’:ab,ti OR FibroScan:ab,ti OR VCTE:ab,ti OR ‘MR elastography’:ab,ti OR MRE:ab,ti OR biopsy:ab,ti OR histologic:ab,ti OR histological:ab,ti OR histopathologic:ab,ti OR histopathological:ab,ti OR fibrosis:ab,ti)	8 Nov 2025	**279**
**Cochrane Library**	(“endoscopic sleeve gastroplasty”:ti,ab OR ESG:ti,ab OR “endoscopic gastric remodeling”:ti,ab OR “endoscopic gastroplasty”:ti,ab OR “bariatric endoscopy”:ti,ab OR “endoscopic bariatric therapy”:ti,ab OR “Apollo OverStitch”:ti,ab OR OverStitch:ti,ab) AND (“metabolic syndrome”:ti,ab OR “metabolic dysfunction”:ti,ab OR “nonalcoholic fatty liver disease”:ti,ab OR “non-alcoholic fatty liver disease”:ti,ab OR NAFLD:ti,ab OR MASLD:ti,ab OR MASH:ti,ab OR NASH:ti,ab OR “hepatic steatosis”:ti,ab OR “fatty liver”:ti,ab OR “steatohepatitis”:ti,ab OR “insulin resistance”:ti,ab OR HbA1c:ti,ab OR “HOMA-IR”:ti,ab OR “FIB-4”:ti,ab OR NFS:ti,ab OR CAP:ti,ab OR “controlled attenuation parameter”:ti,ab OR “MRI-PDFF”:ti,ab OR “liver stiffness”:ti,ab OR elastography:ti,ab OR “transient elastography”:ti,ab OR “shear wave elastography”:ti,ab OR FibroScan:ti,ab OR VCTE:ti,ab OR “MR elastography”:ti,ab OR MRE:ti,ab OR biopsy:ti,ab OR histologic:ti,ab OR histological:ti,ab OR histopathologic:ti,ab OR histopathological:ti,ab OR fibrosis:ti,ab)	8 Nov 2025	**36**
**Scopus (Elsevier)**	TITLE-ABS-KEY(“endoscopic sleeve gastroplasty” OR ESG OR “endoscopic gastric remodeling” OR “endoscopic gastroplasty” OR “bariatric endoscopy” OR “endoscopic bariatric therapy” OR “Apollo OverStitch” OR OverStitch) AND TITLE-ABS-KEY(“metabolic syndrome” OR “metabolic dysfunction” OR “nonalcoholic fatty liver disease” OR “non-alcoholic fatty liver disease” OR NAFLD OR MASLD OR MASH OR NASH OR “hepatic steatosis” OR “fatty liver” OR “steatohepatitis” OR “insulin resistance” OR HbA1c OR “HOMA-IR” OR “FIB-4” OR NFS OR CAP OR “controlled attenuation parameter” OR “MRI-PDFF” OR “liver stiffness” OR elastography OR “transient elastography” OR “shear wave elastography” OR FibroScan OR VCTE OR “MR elastography” OR MRE OR biopsy OR histologic OR histological OR histopathologic OR histopathological OR fibrosis)	8 Nov 2025	**279**
**Web of Science (Core Collection)**	TS=(“endoscopic sleeve gastroplasty” OR “ESG” OR “endoscopic gastric remodeling” OR “endoscopic gastroplasty” OR “bariatric endoscopy” OR “endoscopic bariatric therapy” OR “Apollo OverStitch” OR “OverStitch”) AND TS=(“metabolic syndrome” OR “metabolic dysfunction” OR “nonalcoholic fatty liver disease” OR “non-alcoholic fatty liver disease” OR “NAFLD” OR “MASLD” OR “MASH” OR “NASH” OR “hepatic steatosis” OR “fatty liver” OR “steatohepatitis” OR “insulin resistance” OR “HbA1c” OR “HOMA-IR” OR “FIB-4” OR “NFS” OR “CAP” OR “controlled attenuation parameter” OR “MRI-PDFF” OR “liver stiffness” OR “elastography” OR “transient elastography” OR “shear wave elastography” OR “FibroScan” OR “VCTE” OR “MR elastography” OR “MRE” OR “biopsy” OR “histologic” OR “histological” OR “histopathologic” OR “histopathological” OR “fibrosis”)	8 Nov 2025	**166**

Total records retrieved before deduplication: 855 + 3 References
