# Supplementary material for: Beyond Weight Reduction: An Updated Systematic Review and Meta-Analysis of Metabolic Syndrome Components and Metabolic Dysfunction-Associated Steatotic Liver Disease Outcomes After Endoscopic Sleeve Gastroplasty
Source: J Clin Med. 2026 Aug 27;15(17):6633. doi: 10.3390/jcm15176633 (PMC13566304; doi:10.3390/jcm15176633)
Supplement: Supplementary file 1 [file jcm-15-06633-s001.zip › Supplementary Table S4.pdf]

Supplementary Table S4. Baseline metabolic and liver-related laboratory parameters of the included studies

1st author, year	Follow-up (months)	Baseline-ALT (U/L, mean, ±SD)	Baseline-AST (U/L, mean, ±SD)	Baseline-Hba1c % (±SD)	Baseline HOMA-IR (±SD)	Baseline Triglycerides (mg/dL, mean, ±SD)	BaselineHDL (mg/dL)	Baseline LDL (mg/dL)
Abad 2025 [17]	11 / 17[a]	69.15 (29.03)	53.6 (26.59)	NA	9.06 (4.26)	157 (69.11)	NA	121.68 (30.08)
Abu Dayyeh 2022 [29]	12	NA	NA	5.8 (0.8)	NA	120.8 (63.1)	54.5 (15.5)	115.0 (37.3)
Alexandre 2023 [30]	6, 12	34 (21.32)	27.2 (11.7)	NA	NA	152 (74)	45 (10)	114 (30)
Alqahtani 2022 [31]	6 to 36	NA	NA	NA	NA	NA	NA	NA
Asokkumar 2021 [32][c]	6	68.2 (38.2)	54.6 (27.4)	8 (0.9)	NA	NA	NA	NA
Bhandari 2023 [33]	48	NA	NA	NA	NA	NA	NA	NA
Carr 2022 [34]	6, 12	32.5 (20.1)	26.4 (9.4)	5 (0.3)	NA	124.0 (79.7)	61.9 (19.3)	131.5 (23.2)
Espinet-Coll 2019 [35]	12	27 (23)	24 (11)	6.22 (1.3)	5.4 (6.1)	140 (47)	53 (11)	127 (30)
Hajifathalian 2021 [19][b]	12, 24	29 (16)F, 41(19)M	NA	5.8 (1.2)	6.7 (11)	NA	NA	NA
Jagtap 2021 [36]	12	59.54 (17.02)	78.88 (12.23)	8.71 (1.55)	NA	NA	NA	NA

Jirapinyo 2024 [18]	12	51 (37)	NA	6.7 (1.2)	22.9 (9.2)	NA	NA	NA
Lahooti 2025 [20][b]	12, 36, 60	30.3 (17.3)	NA	5.8 (0.92)	5.8 (11.2)	151.5 (82.8)	NA	130.1 (25.4)

Abbreviations: ALT, alanine aminotransferase; AST, aspartate aminotransferase; F, female; HbA1c, glycated hemoglobin; HDL, high-density lipoprotein cholesterol; HOMA-IR, homeostatic model assessment of insulin resistance; LDL, low-density lipoprotein cholesterol; M, male; Mo, months; mg/dL, milligrams per deciliter; NA, not available; SD, standard deviation; U/L, units per liter. [a]Abad et al. [17] HOMA-IR was assessed at week 48 (~11.1 months); ALT and AST were assessed at week 72 (~16.6 months). That trial does not report high-density lipoprotein cholesterol. See Methods and Table 1 for the prespecified time windows and handling of this study in the pooled analyses. [b]Hajifathalian et al. [19] and Lahooti et al. [20] share the same institution and senior author with overlapping recruitment windows; Hajifathalian et al. was excluded from the HOMA-IR and ALT pooled analyses to avoid double-counting overlapping participants with Lahooti et al. (see Methods and Table 1). [c]Asokkumar et al. [32] reported ALT and AST for the 14 participants with elevated transaminases and fatty liver at baseline, and HbA1c for the 8 participants with type 2 diabetes, rather than for the whole cohort. For Abu Dayyeh et al. [29] the trial reports change from baseline rather than follow-up means; follow-up values used in the analyses were reconstructed as the baseline value plus the published change.
