# Supplementary material for: Beyond Weight Reduction: An Updated Systematic Review and Meta-Analysis of Metabolic Syndrome Components and Metabolic Dysfunction-Associated Steatotic Liver Disease Outcomes After Endoscopic Sleeve Gastroplasty
Source: J Clin Med. 2026 Aug 27;15(17):6633. doi: 10.3390/jcm15176633 (PMC13566304; doi:10.3390/jcm15176633)
Supplement: Supplementary file 1 [file jcm-15-06633-s001.zip › Supplementary Table S5.pdf]

Supplementary Table S5. Complete leave-one-out sensitivity analysis (part 1 of 2): HOMA-IR, ALT, AST, HbA1c and BMI

For each outcome, one study was excluded at a time and the random-effects model (DerSimonian-Laird) was re-run on the remaining studies. MD is expressed as follow-up minus baseline (negative = decrease after ESG). Sig. indicates the 95% CI excludes zero; ns indicates it crosses zero.

HOMA-IR (full analysis: k=4, MD −3.52 [−4.87, −2.18])

Study excluded	MD	95% CI	Significance
Jirapinyo 2024 [18]	−3.48	−5.01, −1.95	Sig.
Espinet-Coll 2019 [35]	−3.92	−5.73, −2.11	Sig.
Abad 2025 [17]	−2.96	−4.01, −1.92	Sig.
Lahooti 2025 [20]	−4.17	−6.41, −1.94	Sig.

ALT (full analysis: k=5, MD −7.82 [−9.69, −5.95])

Study excluded	MD	95% CI	Significance
Alexandre 2023 [30]	−7.90	−9.85, −5.95	Sig.
Carr 2022 [34]	−7.77	−9.66, −5.88	Sig.
Jagtap 2021 [36]	−7.64	−9.56, −5.72	Sig.
Jirapinyo 2024 [18]	−7.78	−9.65, −5.90	Sig.
Lahooti 2025 [20]	−8.98	−13.67, −4.29	Sig.

AST (full analysis: k=3, MD −2.49 [−5.50, 0.53], ns)

Study excluded	MD	95% CI	Significance
Alexandre 2023 [30]	−2.46	−7.69, 2.77	ns

Carr 2022 [34]	−2.05	−5.39, 1.29	ns
Espinet-Coll 2019 [35]	−2.91	−6.18, 0.35	ns

HbA1c (full analysis: k=6, MD −0.33 [−0.47, −0.18])

Study excluded	MD	95% CI	Significance
Abu Dayyeh 2022 [29]	−0.35	−0.57, −0.14	Sig.
Carr 2022 [34]	−0.37	−0.58, −0.16	Sig.
Espinet-Coll 2019 [35]	−0.32	−0.47, −0.17	Sig.
Jagtap 2021 [36]	−0.29	−0.39, −0.19	Sig.
Jirapinyo 2024 [18]	−0.34	−0.51, −0.17	Sig.
Lahooti 2025 [20]	−0.41	−0.63, −0.19	Sig.

BMI (full analysis: k=5, MD −6.21 [−6.31, −6.11])

Study excluded	MD	95% CI	Significance
Alexandre 2023 [30]	−6.20	−6.30, −6.10	Sig.
Alqahtani 2022 [31]	−6.66	−7.34, −5.98	Sig.
Carr 2022 [34]	−6.21	−6.31, −6.11	Sig.
Espinet-Coll 2019 [35]	−6.21	−6.31, −6.11	Sig.
Jagtap 2021 [36]	−6.21	−6.31, −6.11	Sig.

Summary: HOMA-IR, ALT, HbA1c, and BMI are robust to removal of any single study. AST was non-significant in the primary analysis and remained non-significant on exclusion of any single contributing study.
