# Supplementary material for: Beyond Weight Reduction: An Updated Systematic Review and Meta-Analysis of Metabolic Syndrome Components and Metabolic Dysfunction-Associated Steatotic Liver Disease Outcomes After Endoscopic Sleeve Gastroplasty
Source: J Clin Med. 2026 Aug 27;15(17):6633. doi: 10.3390/jcm15176633 (PMC13566304; doi:10.3390/jcm15176633)
Supplement: Supplementary file 1 [file jcm-15-06633-s001.zip › Supplementary Table S6.pdf]

Supplementary Table S6. Complete leave-one-out sensitivity analysis (part 2 of 2): Hepatic Steatosis Index, LDL cholesterol, NAFLD Fibrosis Score, triglycerides and 12-month weight change

For each outcome, one study was excluded at a time and the random-effects model (DerSimonian-Laird) was re-run on the remaining studies. MD is expressed as follow-up minus baseline (negative = decrease after ESG). Sig. indicates the 95% CI excludes zero; ns indicates it crosses zero.

Hepatic Steatosis Index (full analysis: k=5, MD −4.58 [−6.30, −2.86])

Study excluded	MD	95% CI	Significance
Abu Dayyeh 2022 [29]	−5.40	−7.15, −3.66	Sig.
Carr 2022 [34]	−4.22	−5.96, −2.49	Sig.
Espinet-Coll 2019 [35]	−4.07	−5.81, −2.33	Sig.
Hajifathalian 2021 [19]	−5.27	−8.31, −2.24	Sig.
Jagtap 2021 [36]	−4.36	−6.32, −2.40	Sig.

LDL Cholesterol (full analysis: k=5, MD −6.94 [−10.22, −3.65])

Study excluded	MD	95% CI	Significance
Abu Dayyeh 2022 [29]	−7.37	−10.76, −3.97	Sig.
Alexandre 2023 [30]	−6.93	−10.49, −3.36	Sig.
Carr 2022 [34]	−6.62	−9.94, −3.31	Sig.
Espinet-Coll 2019 [35]	−7.04	−10.37, −3.72	Sig.
Lahooti 2025 [20]	−6.21	−12.58, 0.17	ns

NAFLD Fibrosis Score (full analysis: k=4, MD −0.54 [−0.82, −0.26])

Study excluded	MD	95% CI	Significance
Espinet-Coll 2019 [35]	−0.50	−0.84, −0.17	Sig.
Hajifathalian 2021 [19]	−0.73	−1.03, −0.42	Sig.
Jagtap 2021 [36]	−0.40	−0.65, −0.15	Sig.
Jirapinyo 2024 [18]	−0.57	−0.92, −0.21	Sig.

Triglycerides (full analysis: k=5, MD −25.56 [−41.39, −9.73])

Study excluded	MD	95% CI	Significance
Abu Dayyeh 2022 [29]	−31.08	−46.47, −15.68	Sig.
Alexandre 2023 [30]	−23.04	−44.17, −1.91	Sig.
Carr 2022 [34]	−22.77	−39.69, −5.86	Sig.
Espinet-Coll 2019 [35]	−29.93	−45.43, −14.43	Sig.
Lahooti 2025 [20]	−20.89	−40.31, −1.48	Sig.

Weight change, 12 months (full analysis: k=4, MD −16.28 [−18.11, −14.45])

Study excluded	MD	95% CI	Significance
Abu Dayyeh 2022 [29]	−16.78	−18.79, −14.78	Sig.
Alqahtani 2022 [31]	−16.58	−20.82, −12.34	Sig.
Espinet-Coll 2019	−15.99	−16.25, −15.74	Sig.

[35]			
Jagtap 2021 [36]	−16.25	−18.46, −14.04	Sig.

Summary: Hepatic Steatosis Index, NAFLD Fibrosis Score, triglycerides, and weight change at 12 months are robust to removal of any single study. LDL cholesterol loses statistical significance when Lahooti et al. [20] is excluded (95% CI −12.58 to 0.17), indicating this outcome depends on the contribution of the largest included cohort.
