# Supplementary material for: Beyond Weight Reduction: An Updated Systematic Review and Meta-Analysis of Metabolic Syndrome Components and Metabolic Dysfunction-Associated Steatotic Liver Disease Outcomes After Endoscopic Sleeve Gastroplasty
Source: J Clin Med. 2026 Aug 27;15(17):6633. doi: 10.3390/jcm15176633 (PMC13566304; doi:10.3390/jcm15176633)
Supplement: Supplementary file 1 [file jcm-15-06633-s001.zip › Supplementary Table S7.pdf]

Supplementary Table S7. Primary (DerSimonian-Laird) versus sensitivity (Hartung-Knapp-Sidik-Jonkman and paired-correlation) random-effects estimates for each pooled outcome, with approximate 95% prediction intervals for the weight-loss outcomes.

Outcome	k	DL MD (95% CI)	HKSJ MD (95% CI)	I^2 (%)	Paired (r=0.5) MD (95% CI)	I^2 paired (%)	Approximate 95% PI
HbA1c (%)	6	-0.33 (-0.47, -0.18)	-0.33 (-0.52, -0.13)	17	-0.39 (-0.57, -0.21)	55.3	—
HOMA-IR[b]	4	-3.52 (-4.87, -2.18)	-3.52 (-5.66, -1.39)	24	-3.76 (-5.33, -2.19)	52	—
Triglycerides (mg/dL)	5	-25.56 (-41.39, -9.73)	-25.56 (-48.80, -2.32)	66	-23.56 (-39.32, -7.81)	78.3	—
LDL cholesterol (mg/dL)	5	-6.94 (-10.22, -3.65)	-6.94 (-10.85, -3.03)	0	-6.78 (-9.62, -3.95)	7.4	—
HDL cholesterol (mg/dL)	4	3.48 (-1.07, 8.03)	3.48 (-5.85, 12.82)	58	2.90 (-1.58, 7.38)	74.2	—
ALT (U/L)	5[abd]	-7.82 (-9.69, -5.95)	-7.82 (-9.28, -6.36)	0	-7.82 (-9.29, -6.36)	0	—
AST (U/L)	3[ac]	-2.49 (-5.50, 0.53)	-2.49 (-6.32, 1.35)	0	-2.27 (-4.54, -0.01)	0	—
Hepatic Steatosis Index	5	-4.58 (-6.30, -2.86)	-4.58 (-7.44, -1.72)	85	NR (already paired change score)	NR	—
NAFLD Fibrosis Score	4	-0.54 (-0.82, -0.26)	-0.54 (-0.95, -0.13)	32	NR (already paired change score)	NR	—
FIB-4	3	-0.23 (-0.64, 0.18)	-0.23 (-1.13, 0.66)	78	NR (already paired change score)	NR	—
Liver stiffness (kPa)[a]	not pooled	not pooled	not pooled	—	not pooled	—	—
%TBWL, 6 months	3	-16.31 (-17.53, -15.10)	-16.31 (-17.98, -14.65)	0	NR (already paired change score)	NR	NR (df=1)

%TBWL, 12 months	7	-16.54 (-18.60, -14.48)	-16.54 (-20.29, -12.79)	97	NR (already paired change score)	NR	-23.68 to -9.40
Weight change, 12 months (kg)	4	-16.28 (-18.11, -14.45)	-16.28 (-19.35, -13.21)	35	NR (already paired change score)	NR	-24.31 to -8.25
%EWL, 12 months	6	-55.73 (-67.69, -43.76)	-55.73 (-68.70, -42.75)	100	NR (already paired change score)	NR	-97.25 to -14.21
%TBWL, 36 months	3	-14.21 (-20.85, -7.58)	-14.21 (-25.22, -3.21)	100	NR (already paired change score)	NR	NR (df=1)
BMI, 12 months (kg/m²)	5	-6.21 (-6.31, -6.11)	-6.21 (-6.31, -6.11)	0	NR (already paired change score)	NR	-6.56 to -5.86

Signs are expressed as the change from baseline to follow-up. Point estimates are identical between models; the HKSJ adjustment affects only the width of the confidence interval. Liver stiffness was not pooled (see footnote a); the AST estimate did not reach significance under either the DerSimonian-Laird or the HKSJ model. ALT, alanine aminotransferase; AST, aspartate aminotransferase; BMI, body mass index; CI, confidence interval; DL, DerSimonian-Laird; %EWL, percentage excess weight loss; FIB-4, fibrosis-4 index; HbA1c, glycated haemoglobin; HDL, high-density lipoprotein; HKSJ, Hartung-Knapp-Sidik-Jonkman; HOMA-IR, homeostatic model assessment of insulin resistance; k, number of studies; LDL, low-density lipoprotein; MD, mean difference; %TBWL, percentage total body weight loss. For the seven outcomes reported by primary studies as separate baseline and follow-up group means and standard deviations (HbA1c, HOMA-IR, triglycerides, LDL and HDL cholesterol, ALT, AST), the original pooled estimates (DL, HKSJ columns) were calculated treating baseline and follow-up as independent samples, which is conservative but does not use the within-subject (paired) correlation between the two measurements. As a sensitivity analysis, these seven outcomes were re-pooled using the Cochrane Handbook-recommended imputed within-subject correlation (r=0.5) to estimate the standard deviation of the change score, with the follow-up (completer) sample size used as the paired N. Point estimates were materially similar and the direction of every outcome was unchanged; statistical significance was likewise unchanged with one exception, the AST estimate, which is non-significant under the primary model but becomes borderline significant under the paired model (95% CI -4.54 to -0.01) and is flagged as such in the Results; between-

study heterogeneity (I^2) was higher under the paired model, indicating that some of the apparent within-study imprecision under the independent-samples assumption reflects genuine between-study differences once correlation is accounted for. For outcomes reported only as a within-subject mean change, the reported standard deviation (or an imputed correlation of 0.5 when only the standard error of the mean change was available, as described in the Methods) already reflects the paired design, so no further adjustment was applicable (NR). [a]Following the prespecified time-window criterion (12-month bucket = 9-15 months), the sham-controlled trial of Abad et al. [17] was excluded from the ALT, AST and 12-month %TBWL pooled analyses because these outcomes were assessed at week 72 (~16.6 months); it is reported narratively in the main text instead. Liver stiffness was reported by only two studies at genuinely different follow-up windows (12 months and week 72) and is therefore not pooled and reported narratively. [b]Hajifathalian et al. [19] was excluded from the HOMA-IR and ALT rows because of its cohort overlap with Lahooti et al. [20] (same institution and senior author, overlapping recruitment windows; see Methods and Table 1). [c]Lahooti et al. [20], Jirapinyo et al. [18] and Jagtap et al. [36] did not report post-procedural AST values in the original publications and were therefore excluded from the AST pooled analysis; see Supplementary Table S4. [d]Espinet-Coll et al. [35] reported ALT as a median with interquartile range and, in the ESG column, printed values identical to those of the adjacent gamma-glutamyl transferase row, so no usable mean and standard deviation could be recovered; that study was therefore excluded from the ALT pooled analysis. Its triglyceride values were likewise reported as a median with interquartile range, but with identical medians at both timepoints, indicating a symmetrical distribution, and were therefore treated as a mean with standard deviation; this is conservative, because the interquartile range exceeds the corresponding standard deviation and so reduces rather than increases that study's weight. Approximate 95% prediction intervals (PI) were derived for the weight-loss outcomes (the focus of Reviewer comments on Figure 4) using the reported DL mean difference, 95% CI, and k, under the simplifying assumption of equal study weights ($PI = MD \pm t(k-2) \times sqrt(k) \times SE$, where SE is derived from the reported 95% CI); this approximation does not require re-extraction of individual study data and its total variance term is not sensitive to the I^2 split between within- and between-study components, but it becomes uninformative at very low degrees of freedom. The Weight change, 12-month PI (k=4, df=2) is correspondingly wide; the %TBWL, 6-month and %TBWL, 36-month PIs could not be meaningfully approximated (k=3, df=1) and are marked NR. Exact prediction intervals computed from individual study-level data were not derived.
